# Comparing the effects of chemical Ca^2+^ dyes and R-GECO on contractility and Ca^2+^ transients in adult and human iPSC cardiomyocytes

**DOI:** 10.1016/j.yjmcc.2023.04.008

**Published:** 2023-07

**Authors:** Paul Robinson, Alexander J. Sparrow, Yiangos Psaras, Violetta Steeples, Jillian N. Simon, Connor N. Broyles, Yu-Fen Chang, Frances A. Brook, Ying-Jie Wang, Andrew Blease, Xiaoyu Zhang, Yama A. Abassi, Michael A. Geeves, Christopher N. Toepfer, Hugh Watkins, Charles Redwood, Matthew J. Daniels

**Affiliations:** a Division of Cardiovascular Medicine, Radcliffe Department of Medicine, University of Oxford, Oxford, UK; b BHF Centre of Research Excellence, University of Oxford, Oxford, UK; c Agilent Biosciences, Inc., San Diego, CA 92121, USA; d Department of Biosciences, University of Kent, Canterbury, UK; e Department of Genetics, Harvard Medical School, Boston, MA, USA; f Department of Cardiology, Oxford University NHS Hospitals Trust, Oxford, UK; g Department of Cardiovascular Sciences, University of Manchester, Manchester, UK

**Keywords:** Ca2+ indicators, Cardiomyocyte, Contractility, RGECO, Ca2+ handling

## Abstract

We compared commonly used BAPTA-derived chemical Ca^2+^ dyes (fura2, Fluo-4, and Rhod-2) with a newer genetically encoded indicator (R-GECO) in single cell models of the heart. We assessed their performance and effects on cardiomyocyte contractility, determining fluorescent signal-to-noise ratios and sarcomere shortening in primary ventricular myocytes from adult mouse and guinea pig, and in human iPSC-derived cardiomyocytes. Chemical Ca^2+^ dyes displayed dose-dependent contractile impairment in all cell types, and we observed a negative correlation between contraction and fluorescence signal-to-noise ratio, particularly for fura2 and Fluo-4. R-GECO had no effect on sarcomere shortening. BAPTA-based dyes, but not R-GECO, inhibited in vitro acto-myosin ATPase activity. The presence of fura2 accentuated or diminished changes in contractility and Ca^2+^ handling caused by small molecule modulators of contractility and intracellular ionic homeostasis (mavacamten, levosimendan, and flecainide), but this was not observed when using R-GECO in adult guinea pig left ventricular cardiomyocytes. Ca^2+^ handling studies are necessary for cardiotoxicity assessments of small molecules intended for clinical use. Caution should be exercised when interpreting small molecule studies assessing contractile effects and Ca^2+^ transients derived from BAPTA-like chemical Ca^2+^ dyes in cellular assays, a common platform for cardiac toxicology testing and mechanistic investigation of cardiac disease physiology and treatment.

## Highlights

• Chemical Ca^2+^ dyes inhibit contractility in all single cardiomyocytes and reduce force production in loaded cardiac fibres.
• Ca^2+^ dyes inhibit the acto-myosin ATPase with an IC_50_ ∼5μM, competing with the myosin inhibitor blebbistatin (IC_50_ ∼1 μM).
• Ca^2+^ transient visibility in single cells is challenging even at [dye] ∼20 μM.
• R-GECO doesn't impair cellular contractility or acto-myosin ATPase activity, generating clear Ca^2+^ transients at ∼1 μM.
• In small molecule assays compound effects of dye plus drug are apparent and mask the drug effect.

## Graphical abstract

**Figure:**
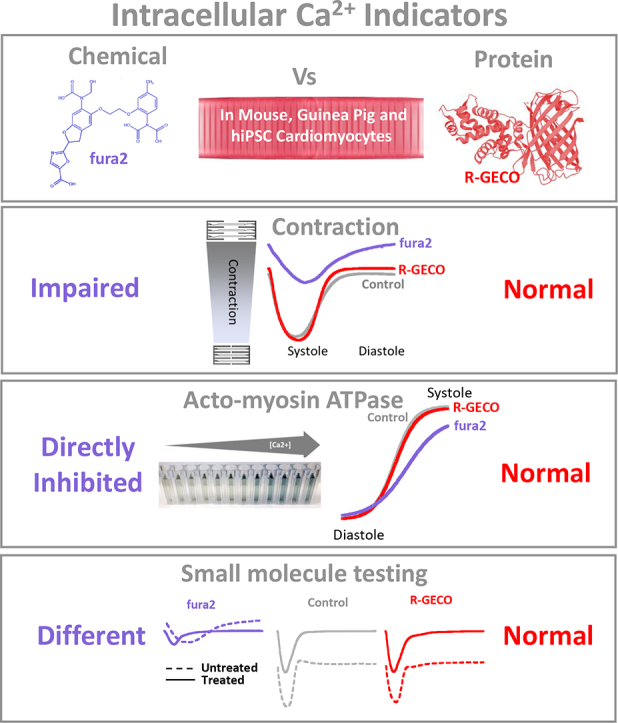

## 1 Introduction

Ca^2+^ regulates contraction in the heart through interplay between extracellular Ca^2+^ sources, intracellular Ca^2+^ stores and buffers. After cardiomyocyte depolarization, extracellular Ca^2+^ enters through voltage gated L-type Ca^2+^ channels held near ryanodine receptors (RyR) in the intracellular membranes of the sarcoplasmic reticulum (SR). RyR channels open triggering an amplifying Ca^2+^-induced Ca^2+^ release (CICR) from the SR before contraction [1]. Ca^2+^ diffuses into the myofilament, binds to troponin C, and activates myosin-ATPase [2]. Ca^2+^ also modulates contractile function via myosin regulatory light chain (RLC) phosphorylation by Ca^2+^/calmodulin kinase (CaMKII) positioned on thick myosin filaments [3]. During relaxation, Ca^2+^ is taken back up into the SR by the sarco-endoplasmic reticulum Ca^2+^ ATPase (SERC2a) and removed from the cell by the plasma membrane Ca^2+^ ATPase (PCMA) or the sodium calcium exchanger (NCX) [ 1], reducing myosin-ATPase activity 100 fold.

Altered Ca^2+^ levels impact cardiac function in disease states like atrial fibrillation, heart failure and cardiomyopathy [4]. Myofilament Ca^2+^ buffering also affects the regulatory system [5]. During systole, up to 90% of intracellular Ca^2+^ binds to TnC [6], and modifying this affinity impacts Ca^2+^ handling and cycling in the cardiomyocyte [7]. Inherited cardiac conditions such as hypertrophic (HCM) and dilated cardiomyopathy (DCM) result from sarcomere gene variants that affect myofilament Ca^2+^ sensitivity and/or contractility [8]. Small molecule modulators that affect contractility and myofilament Ca^2+^ affinity hold potential as therapies for various cardiac conditions [9].

Studying Ca^2+^ handling in cardiovascular research often uses fluorescent chemical Ca^2+^ dyes, such as fura2, Fluo-3/4, and Rhod-2, derived from 1,2-bis(o-aminophenoxy)ethane-N,N,N′,N′-tetraacetic acid (BAPTA), a selective Ca^2+^ chelator discovered by Roger Tsien in 1980 [10]. These dyes carry a negative charge to bind with the positively charged Ca^2+^ cation, making them polar, hence early studies required microinjection [11]. However, AM ester derivatives were developed to enable cell entry by masking the charged tetracarboxylate Ca^2+^ binding motif, which is then cleaved by intracellular esterases to reveal an active Ca^2+^ indicator unable to diffuse out of the cell. Over 2000 primary cardiac research publications have used these dyes [12], including earlier iterations such as Quin2 and Indo-1, affinity variants like Fluo-5 N, and recent derivatives such as the FLIPR® series.

Early use of such compounds in contractile cell models revealed concerns regarding confounding effects “attenuating” contractility with ∼mM range BAPTA concentrations [10]. As these could be overcome by Ca^2+^ this was attributed to Ca^2+^ buffering - if Ca^2+^ is bound to the dye, it is not available to participate in contraction. Dose-dependent reductions in contractile parameters remain with fluorescent BAPTA derivatives such as fura2 [13]. Importantly qualitative differences in the Ca^2+^ transient obtained by chemical dye were noted in cardiac tissue compared to the genetically encoded calcium indicator of the time aqueorin [14], suggesting that when contractile changes happen, the measured Ca^2+^ transient is different. In rat ventricular trabecular preparations, significant reductions in force production were only avoidable at very low fura2 concentrations (2–3 μM, less than twice the auto-fluorescence level) [14]. Changes in both contractility and Ca^2+^ handling are inevitable at higher concentrations [15].

Attributing the contractile impairment observed when using BAPTA and its fluorescent derivatives on Ca^2+^ buffering may be over simplified. Other routes to contractile impairment are known, including the breakdown of the AM-ester and photolytic by-product formation [16], and others mechanisms may exist. For example, in frog skeletal muscle during twitch and tetanic stimulation, the effects of BAPTA-AM are distinct from those of a non-fluorescent Ca^2+^ binder EGTA-AM, which has similar Ca^2+^ buffering activity [17]. Decreased mechanical performance, due to reduced cross bridge formation in the presence of BAPTA, by an unknown mechanism, was suspected [17]. Such effects may impact current work. For example, our studies on HCM and DCM variants in thin filament regulatory proteins showed altered contraction and Ca^2+^ handling via myofilament Ca^2+^ buffering [5,18], but deconvolution of these effects was hindered by the properties of the fura2 dye. To overcome this, we sought alternatives and characterized a genetically encoded Ca^2+^ sensor, R-GECO, introduced by adenovirus, in various cardiomyocyte models [19]. This had no measurable effect on cellular contractility, even when targeted to the myofilament where it produced robust microdomain Ca^2+^ transients revealing HCM variant myofilament buffering directly [20].

Due to significant improvements in optical instrument sensitivity over the past 30 years, which may allow reduced dye concentrations, and the emergence of new model systems (particularly the stem-cell derived cardiomyocyte), we wished to answer the question whether historical shortcomings of chemical Ca^2+^ dyes [[10], [11], [12], [13], [14], [15], [16], [17]] remain relevant in contemporary experimental systems. We particularly wanted to know if they impact experimental outcomes for emerging myocardial therapeutic strategies. Hence, we evaluated BAPTA-derived calcium dyes (fura2, Fluo-4, and Rhod-2, Fig. S1) and genetically encoded Ca^2+^ indicator R-GECO [21] in mouse, guinea pig, and iPSC-derived cardiomyocytes, to determine if contractile impairment due to chemical calcium dye use is still an issue. The acto-myosin ATPase assay was used to investigate mechanistic aspects of the biochemical effects of chemical indicators on contractility. We also examined if contractility modulators such as mavacamten (myosin-ATPase inhibitor) [22], levosimendan (myofilament sensitizer) [23], and flecainide (anti-arrhythmic agent with known off-target effects) [19] give different results depending on the choice of Ca^2+^ indicator using fura2 and R-GECO as exemplars. This study provides a comparison of the suitability of BAPTA-based calcium dyes and R-GECO for evaluating drugs designed to alter contractility.

## 2 Methods

An expanded methods section is available in the Supplementary Material.

### 2.1 Adult cardiomyocyte isolation, contractility, Ca^2+^ transient acquisition and small molecule treatment

Adult left ventricular cardiomyocytes were isolated from wild type 12–16 week old C57Bl6 mice by retrograde perfusion of liberase as previously described [24]. Adult left ventricular guinea pig cardiomyocytes were isolated from 400 g albino guinea pigs by retrograde aortic perfusion of collagenase type II as previously described [5] and cultured in ACCITT_3_ media for 48 h. Freshly isolated mouse or cultured guinea pig cardiomyocytes were treated with 0.2, 1 or 5 μM fura2, Fluo-4, Rhod-2, or SBFI-AM for 5 min at room temperature followed by a 30-min wash period to allow for complete AM ester de-esterification. Mouse / guinea pig cardiomyocytes perfused with Krebs solution containing 1.4 / 1.8 mM CaCl_2_ and paced at 1 / 0.5 Hz respectively using an IonOptix μStep system. Sarcomere shortening was acquired from fast Fourier transform averaging of sarcomeric striations, Ca^2+^ transients were obtained using photomultiplier fluorescence light emission using and ex365/380 em510 or ex488 em525 filters for fura2 or Fluo-4 respectively. To compare sarcomere shortening and fura2 Ca^2+^ transients in the presence of either mavacamten (250 nM), levosimendan (10 μM) or flecainide (0.5 μM), 200 μl of cultured (48 Hours) cardiomyocytes were incubated in either drug or DMSO vehicle for 5 min. At least 3 cell preparations were analysed for each DMSO/drug comparison. No differences or clustering was observed between each cell preparation assessed.

### 2.2 iPSC derived cardiomyocyte culture, SarcTrack and video capture Ca^2+^ transient acquisition

Wild-type iPSC cardiomyocytes with a titin N-terminal enhanced green fluorescent protein (eGFP) tag were kept in culture to passage 35 before differentiation with Wnt pathway modulation with small molecule inhibitors and cultured in in RPMI 1670 / B27 media minus insulin for 7 days. Insulin was added back to the media for 9–11 days until the establishment of spontaneous beating as previously described [25]. Cardiomyocytes were split into 5 groups: DMSO, fura2, Rhod-2, Fluo-4 and SBFI. All chemical indicators were used at 0.2, 1 and 5 μM in Krebs buffer containing 420 μM CaCl_2_. These were applied in low calcium (200 μM) Tyrode-HEPES buffer for 5 min, followed by three 5-min sequential washes with low, medium (300 μM) and high (420 μM) calcium buffer at 37 °C. Imaging was performed with an Olympus IX81 inverted microscope (Olympus, Japan) with an Andor iXon Ultra 897 EMCCD camera (Oxford Instruments, UK). Cell videos were captured at 37 °C at 50 frames per second under electrical pacing at 1 Hz, at 485 / 20–25 nm excitation and 525 / 50 nm emission with at 495 nm dichroic mirror. Videos were processed and parameters extracted with SarcTrack [25]. Ca^2+^ transients were recorded for Rhod-2, Fluo-4. Resultant raw traces were extracted and analysed with CalTrack software [26]. The signal-to-noise ratio was calculated by dividing the peak trace amplitude by the standard deviation of all baseline signal.

### 2.3 In vitro actin activated myosin ATPase assays

Rabbit skeletal myosin S1 ATPase activity was measured in the presence of either isolated rabbit skeletal F-actin or thin filaments reconstituted with actin, human Ala-Ser-tropomyosin, and bovine cardiac troponin as previously described [15]. Assay mixtures were incubated with 0.5 μM fura2, Fluo-4, Rhod-2 or SBFI, inorganic phosphate release was measured calorimetrically after 10 min of incubation at 37 °C as previously described [20].

### 2.4 Cardiac fibre force measurements

Cardiac muscle fibres were taken from 10 week old C57Bl6 mice, demembranated and attached to a 1400A Permeabilized Fibre System from Aurora Scientific as previously described [27]. Following equilibration in pCa 9.0, maximal force was measured using a solution set to pCa 4.0 with addition of 0.2, 1.0 and 5.0 μM fura2 and compared to a 0.2% DMSO control using the movable bath chamber to switch solutions. Total force was corrected for rundown of maximal force at pCa 4.0 at the beginning and end of each experiment and normalised to cross-sectional area of the fibre.

### 2.5 R-GECO recombinant protein production and adenoviral gene transduction into adult and iPSC cardiomyocytes

Recombinant adenovirus expressing R-GECO was generated by Welgen Inc. and used to transduce adult guinea pig or iPSC cardiomyocytes at a multiplicity of infection of 444 or 10 for each cell type respectively. Contractility measurements used either IonOptix or SarcTrack for adult or iPSC cardiomyocytes as described in 2.1, 2.2 respectively. Ca^2+^ transients were acquired from all cell types using 560/25 nm excitation, 620/60 nm emission filters with a 565 nm dichroic mirror and 40 fps video capture imaging at 37 °C using the microscope equipment described in section 2.2. Recombinant R-GECO protein was produced as previously described [20]. 1 μM was added to ATPase assays described in section 2.3. Stopped flow experiments used 3 μM of recombinant protein as previously described [20].

### 2.6 Statistical analysis

All data were assessed for normality using a D'Agostino & Pearson test and, where appropriate, were compared using students *t*-test, or non-parametric Mann-Whitney test for two data sets or One-way ANOVA or non-parametric Kruskal-Wallis test with Tukey post hoc analysis for multiple comparisons where multiple data sets are compared to a single control.

## 3 Results

### 3.1 Chemical Ca^2+^ dyes suppress cardiomyocyte contractile function at concentrations sufficient for Ca^2+^ transients

**Fig. 1:**
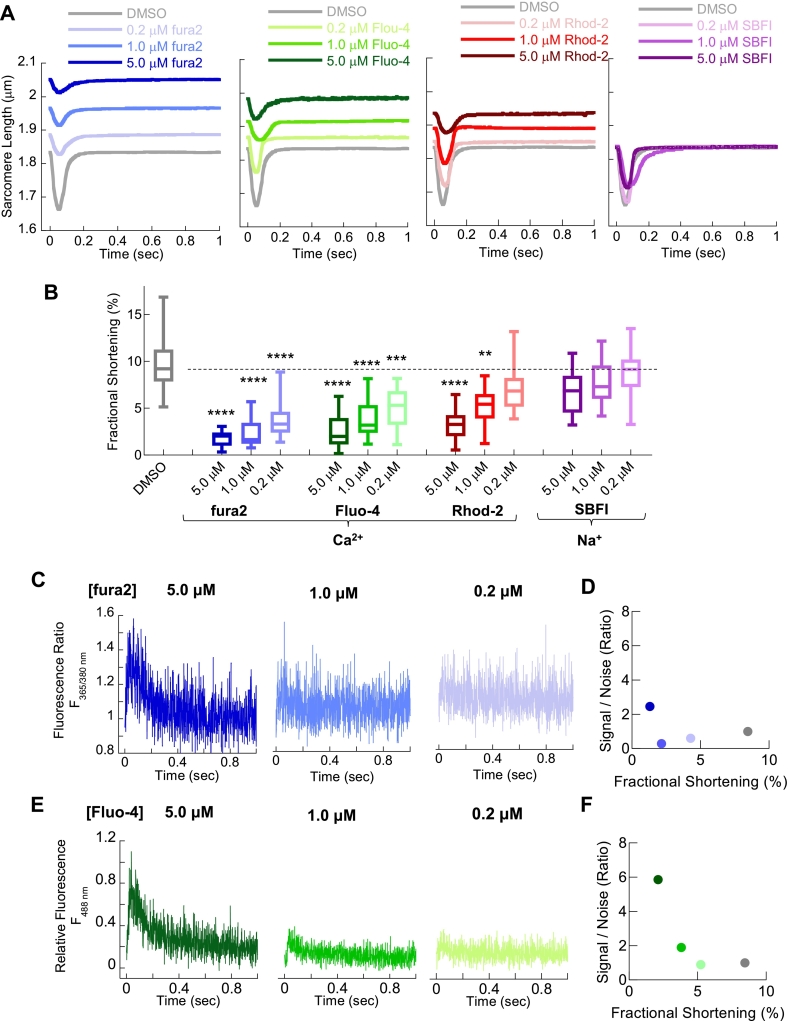
Chemical dyes to measure intracellular Ca^2+^ impair single cell measures of contractility and dose dependent reduction in fluorescence signal to noise ratio in freshly isolated mouse left ventricular cardiomyocytes. Unloaded sarcomere shortening of adult mouse left ventricular cardiomyocytes, paced at 1.0 Hz, were used to test cardiac contractile function in the presence of dyes routinely used in cardiac research on one of the most commonly studied cellular models. Averaged single cell sarcomeric length changes for cells loaded with 0.2 μM, 1.0 μM or 5.0 μM fura2 (Blue); Fluo-4 (green), Rhod-2 (red) and SBFI (purple) are shown in A. Extracted fractional shortening measurements in B in the presence of chemical dyes at the stated concentration. In each case a dose dependent reduction in contraction was observed for all Ca^2+^ indicators. Box and whisker plots give the median average, interquartile range (box) and minimum and maximum data spread (whiskers). * = *p* < 0.05 and ** = *p* < 0.01, *** = *p* < 0.001 **** = *p* < 0.0001 using non-parametric Kruskal-Wallis test (*n* = 37–52 cells from 3 different isolations). Representative raw Ca^2+^ transient traces, collected via photomultiplier photon acquisition at 1000 Hz, are given for fura2 and Fluo-4 in C and E respectively. Average signal to noise ratio extracted from the traces (*n* = 20 separate traces from 3 separate cell isolations) are plotted verses fractional shortening for each indicator in D and E. (For interpretation of the references to colour in this figure legend, the reader is referred to the web version of this article.)

**Fig. 2:**
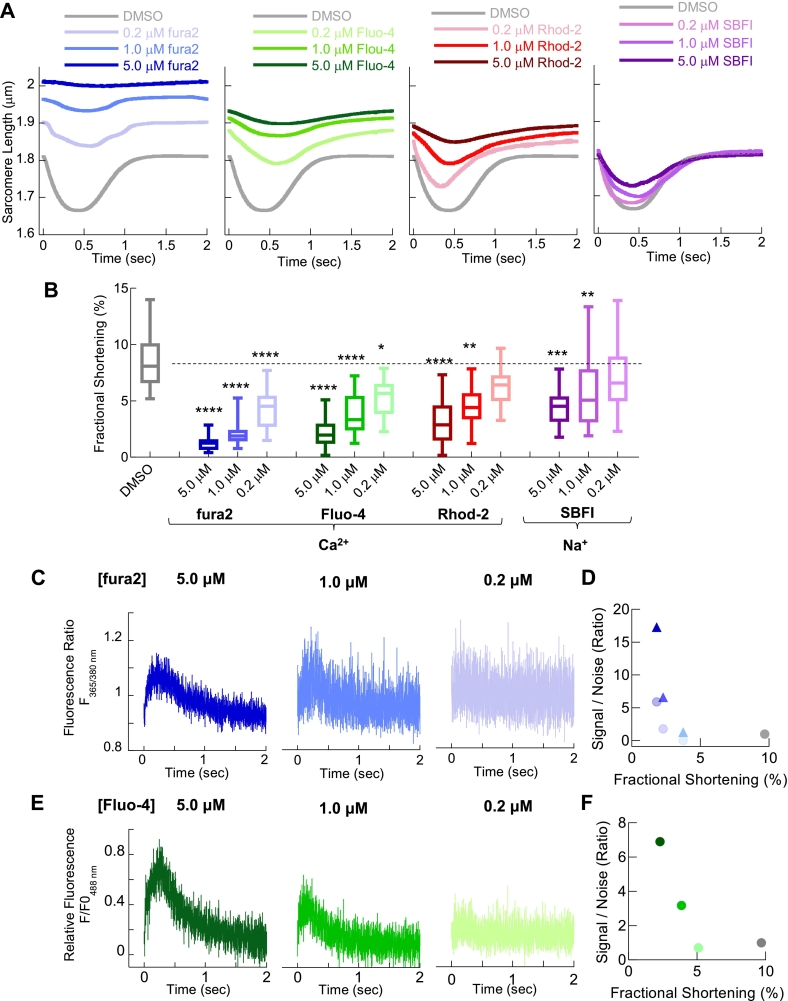
Chemical dyes to measure intracellular Ca^2+^ also blunt contractility in cultured Guinea Pig left ventricular cardiomyocytes and show dose dependent reduction in fluorescence signal to noise. Unloaded sarcomere shortening of cultured (48 h) adult guinea pig left ventricular cardiomyocytes, paced at 0.5 Hz, were used to test cardiac contractile function in the presence of dyes routinely used in cardiac research. Averaged single cell sarcomeric length changes for cells loaded with 0.2 μM, 1.0 μM or 5.0 μM fura2 (Blue); Fluo-4 (green), Rhod-2 (red) and SBFI (purple) are shown in A. Extracted fractional shortening measurements in B in the presence of chemical dyes at the stated concentration. In each case a dose dependent reduction in contraction was observed for all indicators. Box and whisker plots give the median average, interquartile range (box) and minimum and maximum data spread (whiskers). * = *p* < 0.05 and ** = p < 0.01, *** = *p* < 0.001 **** = *p* < 0.0001 using non-parametric Kruskal-Wallis test (*n* = 47–68 cells from 4 different isolations). Representative raw Ca^2+^ transient traces, collected via photomultiplier photon acquisition at 1000 Hz, are given for fura2 and Fluo-4 in C and E respectively. Average signal to noise ratio extracted from the traces (n = 20 separate traces from 3 separate cell isolations) are plotted verses fractional shortening for each indicator in D and E. (For interpretation of the references to colour in this figure legend, the reader is referred to the web version of this article.)

We tested the dose-dependent effects of fura2, Fluo-4, and Rhod-2 on sarcomere shortening in both mouse and guinea pig ventricular cardiomyocytes, allowing comparisons in cardiomyocytes containing both fast α-myosin (mice, Fig. 1) and slow β-myosin (guinea pig, Fig. 2) isoforms. Brief dye loading (5 min) at typical concentrations (0.2, 1, and 5 μM) resulted in a significant, dose-dependent reduction in fractional shortening ((Fig. 1A and B and Fig. 2A and B), with 5 μM fura2 reducing fractional shortening by 81.0 ± 12.4% (*n* = 52, *p* < 0.0001) and 87.1 ± 12.3% (*n* = 54, p < 0.0001) compared with control in mouse and guinea pig cardiomyocytes, respectively. SBFI, a Na^+^ indicator used as a control for the toxic by-products of AM-de-esterification, had no significant effect on fractional shortening in mouse cardiomyocytes and only a limited effect in guinea pig cardiomyocytes. While we observed dose-dependent lengthening of the sarcomere with each Ca^2+^ dye tested, only higher concentrations of Fluo-4 and Rhod-2 slowed cellular relaxation of mouse cardiomyocytes, but in contrast, relaxation of guinea pig cells was slowed by all dyes (Fig. S2A—D). Signal-to-noise ratios of fura2 and Fluo-4 fluorescence increased with dose (Fig. 1C and E and Fig. 2 C and E respectively) but were inversely correlated with fractional shortening ((Fig. 1D and F and Fig. 2 D and F). Lower dye concentrations retained contractile impairment but did not deliver sufficient signal-to-noise and were unsuitable for accurate processing by commercially available analysis tools (IonWizard) or our own open-source software (CalTrack). At lower concentrations, this was not improved by averaging transient data to boost signal-to-noise, for example, averaging 10 transients did not improve signal-to-noise sufficiently (Fig. S3).

### 3.2 Chemical Ca^2+^ dyes have a milder effect on contractile function in cultured iPSC derived cardiomyocytes

**Fig. 3:**
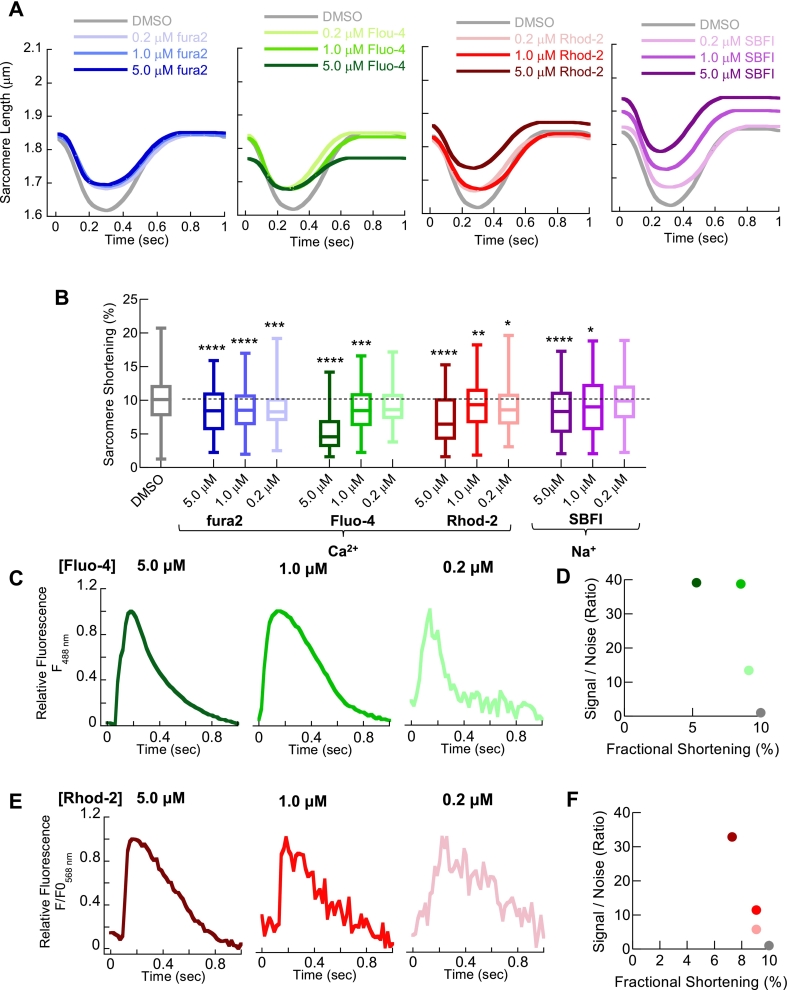
Chemical dyes Ca^2+^ dyes impair single cell measures of contractility in cultured human iPSC derived cardiomyocytes to a lesser extent with improved fluorescence signal to noise using high frame rate video acquisition. Sarcomere shortening of cultured human iPSC derived cardiomyocytes, paced at 1.0 Hz using SarcTrack software to measure individual sarcomere movement and correct for orientation differences of the myofilaments. We tested the same chemical dyes and loading times as Fig. 1, Fig. 2. Simulated curves from average extracted parameters for cells loaded with 0.2 μM, 1.0 μM or 5.0 μM fura2 (Blue); Fluo-4 (green), Rhod-2 (red) and SBFI (purple) are shown in A. Extracted fractional shortening measurements in B in the presence of chemical dyes at the stated concentration. In each case a degree of dose dependent reduction in contraction was observed for all indicators. Box and whisker plots give the median average, interquartile range (box) and minimum and maximum data spread (whiskers). * = p < 0.05 and ** = p < 0.01, *** = p < 0.001 **** = *p* < 0.0001 using non-parametric Kruskal-Wallis test. (*n* = 198–1321 cells from 4 different plate seedings). Representative raw Ca^2+^ transient traces, collected by video acquisition at 50 fps, are given for Fluo-4 and Rhod-2 in C and E respectively. Average signal to noise ratio extracted from the traces (*n* = 20 separate traces from 3 separate cell seedings) are plotted verses fractional shortening for each indicator in D and E. (For interpretation of the references to colour in this figure legend, the reader is referred to the web version of this article.)

Patient-derived induced pluripotent stem cell (iPSC) or gene-edited human embryonic stem cell-derived cardiomyocytes can be used for human disease modelling and therapeutic screening. Although iPSC cardiomyocytes respond to electrical pacing and provide readouts of contractility and calcium cycling [28], their structural maturation is limited, resulting in reduced force production and resting sarcomere length compared to adult ventricular myocytes [29]. We used SarcTrack [25] to evaluate the effect of three chemical Ca^2+^ dyes and SBFI on cellular contractility in iPSC cardiomyocytes stimulated at 1 Hz. All Ca^2+^ dyes tested significantly impaired contractility, with 5.0 μM Fluo-4 reducing fractional shortening by 47.1 ± 15.2% (*n* = 670, *p* < 0.0001) compared to untreated controls (Fig. 3A and B). SBFI also reduced fractional shortening by 16.0 ± 2.6% (*n* = 198, p < 0.0001) at 5 μM. The signal-to-noise ratio was assessed across multiple doses of Fluo-4 and Rhod-2 (Fig. 3C-F). In contrast to the primary rodent cardiomyocytes observed in a photometric system, low indicator concentrations (0.2 μM or 1.0 μM) in this model were detectable by EMCCD cameras, although automated analysis using CalTrack [26] remains impaired by the low signal-to-noise.

### 3.3 Chemical Ca^2+^ dyes directly inhibit in vitro actin activated acto-myosin ATPase activity and force development in cardiac acto-myosin

**Fig. 4:**
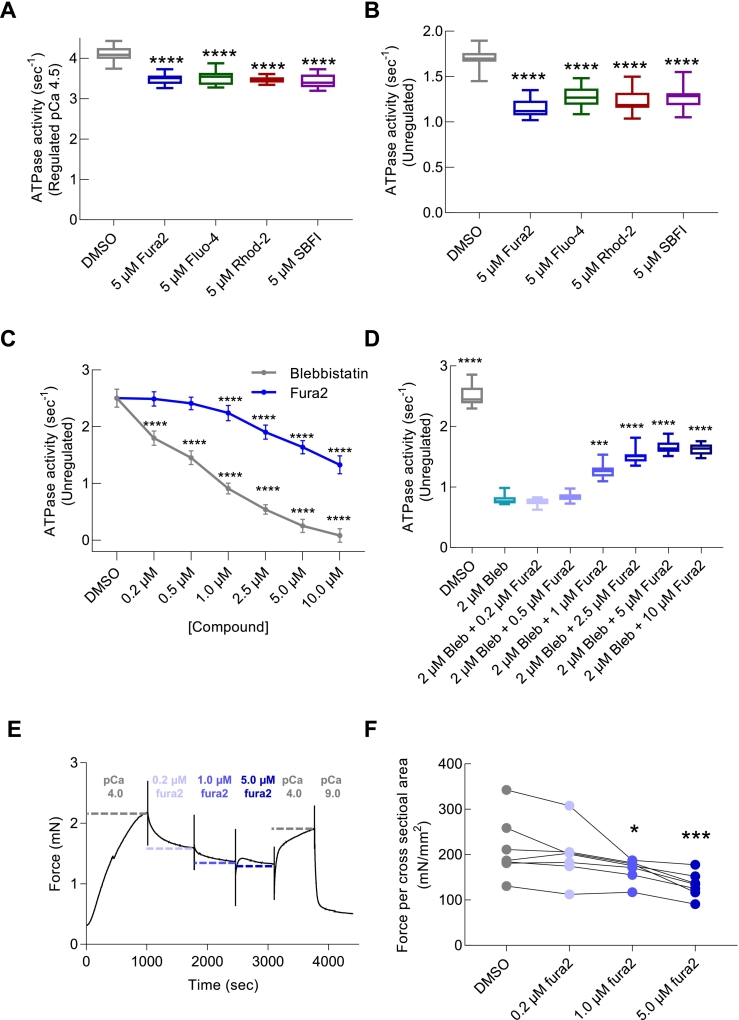
Chemical dyes directly reduce in vitro acto-myosin ATPase and force in permeabilised cardiac fibre preparations The action of a range of chemical dyes on myofilament function was assessed using in vitro actin activated acto-myosin S1 ATPase assays. The maximally activated (pCa 4.5) ATPase of reconstituted myofilaments is inhibited in the presence of fura2-AM ester (5.0 μM, Blue), Fluo-4-AM ester (5.0 μM, green) or Rhod-2-AM ester (5.0 μM, red) (*n* = 16 separate preparations of the assay), B. Reductions in maximal ATPase activity of similar magnitude are also apparent when the thin filament components are not present in reactions containing only actin and the S1 myosin head (n = 16 separate preparations of the assay). C, fura2 has a higher K_d_ compared to blebbistatin for the inhibition of unregulated acto myosin ATPase. In a competition assay (D) using increasing concentrations (0.2–10 μM) of fura2 with a fixed concentration of blebbistatin (2 μM), higher fura2 concentrations blocked the inhibitory effect of blebbistatin (*n* = 16 separate repetitions of the assay). E, shows an example raw trace reporting the effect of increasing concentrations of fura2 (0.2, 1.0 and 0.5 μM) on force development in mouse left ventricular muscle fibres in high Ca^2+^ (pCa 4.0), at 2.2 nm sarcomere length. F shows that 5.0 μM fura2 significantly reduces maximally activated force (*n* = 7 fibres from 3 separate heart dissections). Box and whisker plots B, C, D and E give the median average, interquartile range (box) and minimum and maximum data spread (whiskers). * = *p* < 0.05, *** = *p* < 0.001 and **** = *p* < 0.0001 using one way ANOVA for ATPase assays and paired Friedman test for force measurements. (For interpretation of the references to colour in this figure legend, the reader is referred to the web version of this article.)

To determine if the impaired contractility in cardiomyocyte models is due to calcium buffering or other cross-bridge dependent mechanisms, we assessed the in vitro acto-myosin ATPase activity, an in vitro correlate of contraction [30], in the presence or absence of Ca^2+^ dye (5 μM) using bovine cardiac isoforms of actin, myosin, and troponin complex, along with human recombinant Ala-Ser α-tropomyosin where the additional di-amino acid modification mimics endogenous N-terminal acetylation [31]. The rate of ATP hydrolysis was measured under maximally activating calcium (pCa 4.5) for previously assessed chemical dyes (Ca^2+^, Na^+^) at 5 μM concentrations. All dyes reduced ATPase rates, with fura2-AM, Fluo-4-AM, Rhod-2, and SBFI-AM causing reductions of 15.3 ± 0.8%, 14.4 ± 1.1%, 15.8 ± 0.5%, and 16.4 ± 1.1%, respectively (*n* = 16, *P* < 0.0001 for all) (Fig. 4A).

To determine if the inhibition mechanism was due to competition for Ca^2+^ by the thin filament regulatory components or directly via actin/myosin, we repeated the assay without the thin filament regulatory proteins. Chemical dyes directly inhibited acto-myosin ATPase in all cases, with reductions of 32.4 ± 1.5% for fura2-AM, 25.4 ± 1.7% for Fluo-4-AM, 27.5 ± 1.8% for Rhod-2-AM, and 25.7 ± 1.88% with SBFI-AM (all n = 16, *p* < 0.0001) (Fig. 4B). The ATPase assays were repeated using regulated thin filaments and a fura2 de-esterified free salt to confirm that inhibition arose from the active indicator via the core BAPTA structure. We observed consistent reductions in EC_50_ and ATPase rate at pCa4.5 in the presence of the active Ca^2+^ indicator (Fig. S4). Intracellular fura2 levels were determined in guinea pig cardiomyocytes to ensure that the in vitro dye concentrations reflected the intracellular dye concentrations achieved in intact cell studies. We found that intracellular fura2 levels were 7 to 20-fold higher than the exogenous cell concentration treated (5 and 1 μM respectively) after 5 min of dye exposure (Fig. S5A—D). This suggests that cellular accumulation of dye may expose myofilaments to concentrations higher than those tested in vitro.

To compare the effect of Ca^2+^ dyes on acto-myosin inhibition to a known inhibitor (blebbistatin) [32], we performed unregulated acto-myosin ATPase assays with a range of fura2 and blebbistatin concentrations ((0.2–10.0 μM). The IC_50_ for blebbistatin was 0.88 ± 0.04 μM, and 4.92 ± 0.62 μM for fura2-AM (Fig. 4C). To determine if fura2 used the same inhibitory site as blebbistatin, we performed a competition assay using a fixed 2 μM (double the IC_50_) concentration of blebbistatin. Progressive prevention of blebbistatin inhibition between 0.2 and 2.5 μM fura2 was seen, followed by a plateau (Fig. 4D).

Finally, using skinned cardiac fibres from mouse left ventricle to measure the effect of fura2 on the development of maximally activated force (pCa 4.0) at an isometrically stretched sarcomere length of 2.2 μm we tested if dye inhibition of acto-myosin interaction was preserved in a co-operative system under load. Sequential increase in fura2 to concentrations of 0.2, 1.0 and 5.0 μM reduced the force reading acquired (Fig. 4E). Both 1 and 5 μM of fura2 significantly reduced the maximal force/cross-sectional area compared to a DMSO control (*n* = 7, Δ-Force = −49.6 ± 9.3 and − 80.2 ± 10.3 mN.mm2 *p* < 0.05 and < 0.001 respectively) (Fig. 4E, F).

### 3.4 The genetically encoded Ca^2+^ sensor R-GECO does not affect cellular contractility or in vitro acto-myosin ATPase activity

Proteins have been developed to measure Ca^2+^ with advantages of subcellular targeting, affinity and spectral variants that complement the chemical dye toolset [12]. The genetically encoded Ca^2+^ indicator R-GECO is a red spectral variant of a circularly permuted fluorescent reporter protein (mApple) engineered to contain a classic Ca^2+^ binding domain (Calmodulin/M13) [21]. It can be expressed in primary cell models using adenoviral gene transduction, but ∼48 h are needed for indicator production and maturation. While use in rapidly de-differentiating models like mouse cardiomyocytes requires transgenic or in vivo viral transduction steps [19], R-GECO can be used in more stable guinea pig and iPSC cardiomyocyte models revealing beat-to-beat Ca^2+^ transients [19,20].

**Fig. 5:**
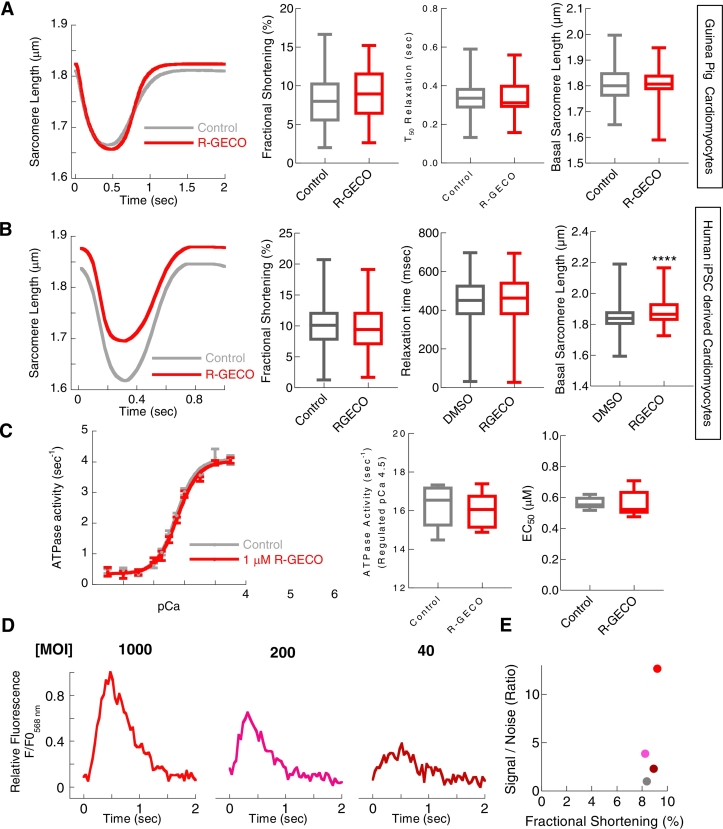
R-GECO has no measurable effects on contractility in adult ventricular cardiomyocytes and only a small increase in basal sarcomere length in hiPSCs, with no changes to acto-myosin ATPase in vitro, Ca^2+^ transients also show improved signal to noise. Unloaded sarcomere shortening measurements comparing uninfected with R-GECO transduced (MOI 1000) cardiomyocytes showed no alterations to the fractional shortening or relaxation times (adjacent plots) A (*n* = 52 cells from 3 separate cell isolations). Simulated curves derived from extracted parameters from SarcTrack software were used to plot sarcomere length changes in paces (1 Hz) hiPSC cardiomyocytes in B (*n* = 1321 cells for control and 198 cells from R-GECO transduced preparations, each from 3 separate cell seedings). 1.0 μM R-GECO was added to the reconstituted acto-myosin ATPase components without clear reduction in maximal ATPase activity or shift in EC_50_ (adjacent plots) C (*n* = 6 separate assays). Example raw Ca^2+^ traces (F_581_) using high frame rate video acquisition at 25 Hz, for guinea pig cardiomyocytes transduced with R-GECO at MOIs of 1000, 200 and 40 are given in D. Pairwise sarcomere shortening magnitudes from were taken from A and plotted vs signal to noise ratios derived from the dynamic signal, F_581_, E (*n* = 20 separate traces). Box and whisker plots give the median average, interquartile range (box) and minimum and maximum data spread (whiskers). **** = p < 0.0001 using unpaired student *t*-tests.

In contrast to the chemical Ca^2+^ indicators (Fig. 2, Fig. 3), transduction of R-GECO into guinea pig or iPSC cardiomyocytes did not significantly affect contractility (Fig. 5A and B), except for a possible small (<0.1 μm) alteration to basal sarcomere length in iPSC cardiomyocytes (1.75 ± 0.32% (*n* = 1321 for control cells, 198 for R-GECO transduced, *p* < 0.0001). Additionally, there was no difference in cardiomyocyte contractility between guinea pig cardiomyocytes transduced with R-GECO and those infected with a control adenovirus expressing GFP, or when compared to uninfected control cells *(*Fig. S6*)*, ruling out significant contractile effects induced by adenoviral infection itself. We observed MOI-dependent expression variability of genetically encoded indicators following viral transduction, typically 0.2–2 μM range (Fig. S5E and F), with an estimated average expression of ∼1 μM in guinea pig cardiomyocytes [20]. Unlike the chemical dyes (Fig. 4A) in the in vitro acto-myosin ATPase assay, we found no difference to EC_50_ at maximal (pCa 4.5), or minimal (pCa 8.5), activated ATPase activity in the presence of R-GECO (Fig. 5C). In Ca^2+^ transient assessment, the R-GECO signal-to-noise was better (Fig. 5D-E) than that obtained with chemical Ca^2+^ dyes, with contractility maintained irrespective of the expression levels tested (Fig. 5E).

Finally, we performed dual indicator Ca^2+^ imaging [20], where cells expressing a fixed concentration of (red) R-GECO (MOI = 400 or ∼ 1 μM) were incubated with increasing concentrations of (green) Fluo-4. We observed a dose-dependent reduction of R-GECO fluorescent signal, of up to 66.4 ± 2.25% (*n* = 28) for cells loaded with 5 μM Fluo-4 (Fig. S7A—D), indicative of the stronger Ca^2+^ affinity of the dye as expected. The kinetics and fluorescent intensity of Fluo-4 was unaffected by the presence of R-GECO (Fig. S7E and F). Collectively, our results suggest that R-GECO reports the Ca^2+^ transient with better SNR, less dose dependency, less contractile impairment, and less Ca^2+^ buffering than chemical dyes in the studied cardiomyocyte models.

### 3.5 Comparison of contractile function in fura2 or R-GECO treated guinea pig cardiomyocytes treated with mavacamten, levosimendan or flecainide

Concerns about the use of chemical dyes as Ca^2+^ indicators, which cause contractile impairment (and reveal aberrant Ca^2+^ transients in those settings) could be significant for the development of novel therapeutics that work at the myofilament [9]. There is clear potential for synergistic or antagonistic effects of the experimental compound with those identified to be a general feature of the commonly used chemical dyes (Fig. 1, Fig. 2, Fig. 3, Fig. 4). The overall impact on the Ca^2+^ signal is uncertain, but could lead to safe compounds being rejected, or harmful compounds being accepted.

To investigate this potential issue, we tested the suitability of both chemical and genetic Ca^2+^ indicators (fura2 and R-GECO, respectively) for evaluating the effects of three representative drugs that affect both contractility and Ca^2+^ transients. These drugs included mavacamten [22], which suppresses contractility, levosimendan [23], which improves contractility, and flecainide [19], which has complex effects on ion channels and contractility that ultimately prolong the Ca^2+^ transient and cause negative inotropy [[33], [34], [35]].

**Fig. 6:**
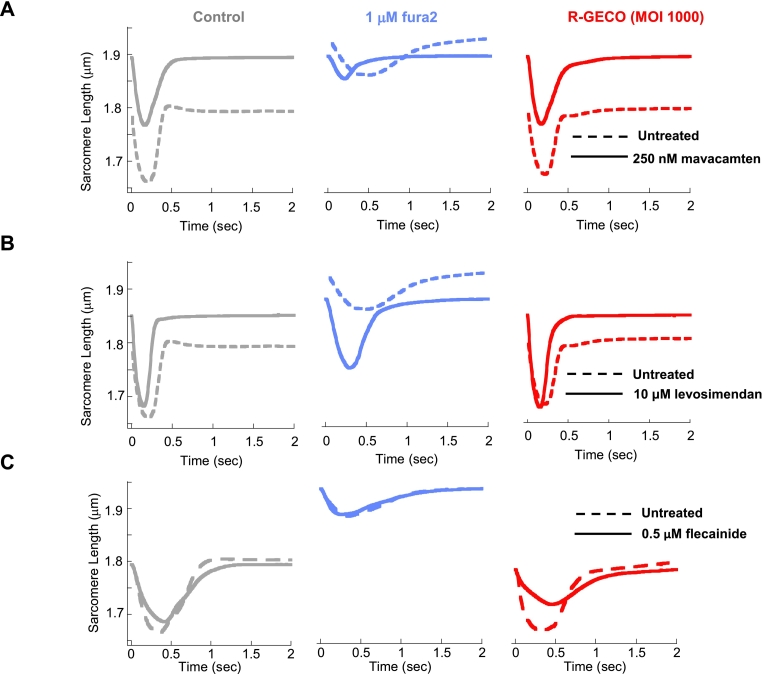
The dye dependant effect of 250 nM mavacamten, 10 mM levosimendan or 0.5 mM flecainide on adult GPCMs. Averaged curves from control (dashed line) or drug exposed (solid line) are given for each of the stated indicator, and small molecule combinations: 250 nM mavacamten A, 10 mM levosimendan B or 0.5 mM flecainide C. Due to variability inherent in primary cell preparations controls are presented for each condition and the sample split for paired analysis with drug. Absolute numbers for extracted parameters and *n* for mavacamten, levosimendan and flecainide treatment are plotted and tabulated in (Fig. S8, S9 and S10) respectively. (*n* = 18–36 cells from 3 separate isolations).

We investigated the effects of these drugs on adult guinea pig cardiomyocyte contractility with and without each Ca^2+^ reporter (Fig. 6, and Figs. S8–10). Mavacamten (250 nM) increased sarcomere length (5.51 ± 0.39% *p* < 0.001) and decreased T50 relaxation time (15.1 ± 5.4 msec *p* < 0.05) without attenuating fractional shortening (3.7 ± 6.7%, n/s) (Fig. 6A, and Fig. S7). The presence of R-GECO makes little difference, with the exception that the small change to T_50_ relaxation was not seen. However, the addition of 1 μM fura2 (the lowest dose from which we can identify a Ca^2+^ transient (Fig. 2C), a fifth of the IC_50_ Fig. 4C) with mavacamten significantly worsened all contractile parameters measured, for example fractional shortening falls by 44 ± 10.7% (p < 0.001) (Fig. S8).

Levosimendan (10 μM) increased fractional shortening in all experimental groups, but only decreased basal sarcomere length in fura2-treated cells (−2.64 ± 0.75%) giving the false impression of hypercontraction, rather than the relaxation shown in the control or R-GECO transduced cardiomyocyte (+3.23 ± 0.95% and + 2.06 ± 0.85% respectively) (Fig. 6B and Fig. S9).

Flecainide (0.5 μM) appears to have no contractile effect in the presence of fura2, yet in control and R-GECO treated cells fractional shortening is reduced (by 20.9 ± 4.4% *p* < 0.01) and contraction and relaxation times increase (by 28.4 ± 11.2% p < 0.05 and 24.5 ± 6.9% p < 0.01 respectively) (Fig. 6C and Fig. S10).

### 3.6 Comparison of Ca^2+^ transient alterations in fura2 or R-GECO treated guinea pig cardiomyocytes treated with mavacamten, levosimendan and flecainide

Having established that contractile properties change when dye and drug are present, we must recognise that this is an artificial concern as contractile measurements do not require a Ca^2+^ dye. However, a calcium reporter is required for parallel safety and efficacy studies for drugs, or understanding genetic variants that may cause disease through Ca^2+^ handling [5]. Before beginning to test whether the Ca^2+^ dyes can alter to Ca^2+^ signals, we firstly measured the Ca^2+^ binding kinetics of R-GECO to ensure its suitability to report beat-to-beat variations in Ca^2+^ transient behaviour.

At 3 μM Ca^2+^ R-GECO had a k_on_ of 4.59 × 10^−4^ M^−1^ s^−1^ and k_off_ of 17.2 s^−1^ which gave a relaxation time (1/(k_on_ + k_off_)) of ∼5.8 msec at 37 °C and pH 7.4 (Fig. S11). Although association and dissociation rates are intrinsically slower than fura2 (k_on_ of 6.03 × 10^−8^ M^−1^s^−1^; k_off_ of 97.8 s^−1^) [36] over the physiological pacing range (1–3 Hz) Ca^2+^ signal readouts are not constrained by the properties of the probe itself, making it a viable alternative to chemical Ca^2+^ dyes for safety and efficacy studies of drugs.

**Fig. 7:**
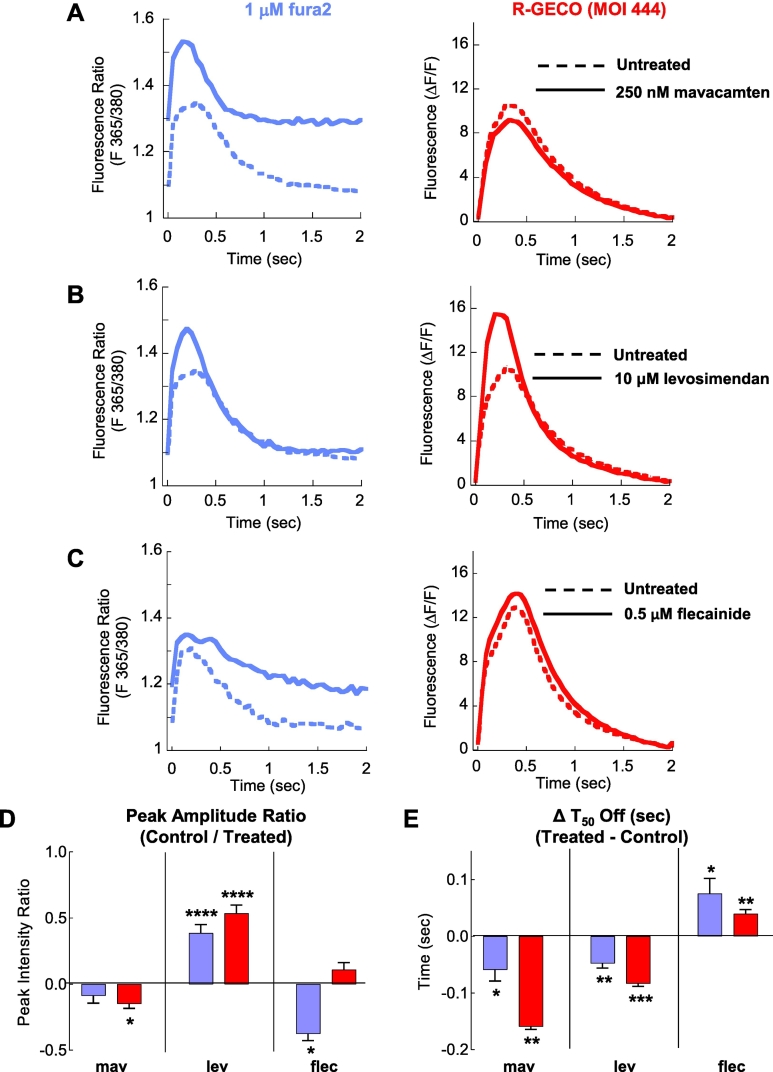
The dye dependant effect of myofilament and Ca^2+^ modulating compounds on adult guinea pig cardiomyocyte Ca^2+^ transient measurements. The effects of myofilament and intracellular Ca^2+^ modulating compounds on Ca^2+^ transients were measured in electrically paced (0.5 Hz) adult guinea pig left ventricular cardiomyocytes. The Ca^2+^ dependent dynamic signals of 1 μM fura2 (F_365/380_) (external loading concentration) and R-GECO (F_581_) (MOI 444) for 250 nM mavacamten, 10 μM levosimendan and 0.5 μM flecainide are shown in A, B and C respectively. Each comparison is made in paired experiments between drug treated (solid lines) and DMSO control treated (dashed lines) for fura2 loaded (purple), and R-GECO infected (red). Extracted parameters are tabulated in Table S1. Changes in peak amplitude ratio and ΔT_50_ off are shown for fura2 and R-GECO containing cells (including SEM and *p* values) for all compounds (mav, lev and flec) are plotted in D and E respectively. Error bars are SEM, * = p < 0.05 ** = *p* < 0.01, *** = p < 0.001 and **** = p < 0.0001 using non-parametric Mann-Witney tests to compare untreated to treated cells (*n* = 22–84 cells from 3 separate isolations). (For interpretation of the references to colour in this figure legend, the reader is referred to the web version of this article.)

When 250 nM mavacamten was applied to fura2-treated cells, it caused a significant increase in basal Ca^2+^ levels, which cannot be assessed by the intensiometric R-GECO (Fig. 7A; Table S1). Conversely, a small reduction in Ca^2+^ transient amplitude was detected by R-GECO, but this was not detected in fura2 loaded cells (Fig. 7D).

However, levosimendan (10 μM) increased Ca^2+^ transient amplitude and the speed of Ca^2+^ reuptake irrespective of the Ca^2+^ measurement method (Fig. 7B, D and E). In contrast, flecainide had subtle effects on Ca^2+^ transients that were difficult to interpret. For example, in the presence of fura2, flecainide increased diastolic Ca^2+^, reduced Ca^2+^ transient amplitude, and prolonged Ca^2+^ reuptake without a net effect on contractility. In R-GECO-treated cells, flecainide did not alter the Ca^2+^ transient amplitude, but did prolong relaxation (Fig. 7C-E; Table S1).

The similarities and differences of the two indicators effects on contractility (Fig. 6) and Ca^2+^ transients (Fig. 7) are perhaps best demonstrated by plotting sarcomere length verses indicator fluorescence using the common time domain to both measurements (Fig. S12). Contractile impairment clearly alters the shape and size of the loop plots derived from fura2 treated cardiomyocytes compared to R-GECO. They also illustrate the improved resolution of measuring drug effect changes to contractility and Ca^2+^ when using a reporter which does not impair contraction. Subtle effects are more likely to be detected, and potentially spurious toxic read-outs may be avoided.

## 4 Discussion

In this study, we tested widely used chemical indicators to assess cardiac Ca^2+^ handling, comparing their effect on contractility to a Na^+^ indicator negative control and a genetically encoded alternative Ca^2+^ indicator R-GECO. We assessed contractility and Ca^2+^ handling in multiple cardiomyocyte models and uncovered a novel inhibitory mechanism from in vitro ATPase and force generation of skinned cardiac fibre assays. Our study provides mechanistic insights into the confounding effects and usefulness of these indicators, which can guide future experiments on cardiovascular excitation-contraction coupling.

### 4.1 Mechanistic insights of contractile impairment due to chemical Ca^2+^ dyes

We found that BAPTA-derived indicators cause contractile impairment in cardiac cell models due to multiple components not present in genetically encoded sensors. A major aspect is Ca^2+^ buffering; an issue of indicator concentration and Ca^2+^ binding affinity. Chemical dyes reach higher intracellular concentrations (33.5 ± 8.1 and 19.9 ± 5.7 μM, for 5 μM and 1 μM loading over 5 min) (Fig. S5A—D), than virally transduced GECIs (∼0.2–2 μM range at 48 h (Fig. S5E and F)). Indeed historical literature [13] shows that when intracellular dye concentrations of 2–3 μM (i.e. half the measured IC_50_ (Fig. 4C), equivalent to R-GECO expression levels) are obtained by microinjection contractile impairment is avoided. Unfortunately, microinjection is not scalable. Furthermore, BAPTA-derived dyes have lower steady state binding constants (fura2: 145 μM [16], Fluo-4: 345 μM [37], Rhod-2: 370 μM [38]) which out compete R-GECO (482–860 μM [20,21]) for available Ca^2+^ (Fig. S7A—D).

The toxic by-products of chemical dye loading are the AM-ester breakdown products such as formaldehyde and acetic acid which affect protein conformation [39] and lower intracellular pH. Lower pH can inhibit acto-myosin contractility and affect maintenance of normal Ca^2+^ handling and membrane potential [40]. The results of the control indicator for AM-ester effects (SBFI) suggest this alone has limited importance (Fig. 1, Fig. 2, Fig. 3; and Fig. S2). Furthermore, the role of phototoxic by-products [16] can be excluded as in-incubator impedance assays of iPSC cardiomyocytes captures dose dependent contractile inhibition in the absence of extrinsic excitation light required for fluorescence recordings (Fig. S13).

BAPTA-derived chemical indicators have been reported to directly inhibit cardiomyocyte sodium/potassium ATPase activity [41]. Although prior work hinted at a mechanism of contractile impairment beyond Ca^2+^ buffering [17] direct inhibition of the acto-myosin ATPase was unexpected. The fura2/blebbistatin competition ATPase assay indicates a similar mechanism of inhibition conferred by the chemical dyes, and almost equivalent potency (IC_50_ for blebbistatin was 0.88 ± 0.04 μM, and 4.92 ± 0.62 μM for fura2-AM (Fig. 4C)). Blebbistatin allosterically stabilizes ATP binding to the catalytic cleft of myosin and prolongs ADP release reducing ATPase activity independently of Ca^2+^ sensitive regulation of the actin filament [32]. As acto-myosin generated force is suppressed in ordered co-operative muscle fibres following incubation with fura2, we expect dye use could affect functional measurements in assay systems under load such as engineered heart tissue, intact muscle strips, and whole working heart preparations used for optical mapping. Since the in vitro acto-myosin ATPase has almost equivalent results (Fig. 4A-B) for different spectral dye variants, the inhibition is likely due to the core BAPTA element, rather than the variable fluorescent adduct, and so will be difficult to avoid.

As dyes accumulate irreversibly once de-esterified [42]; intracellular concentrations exceed the extracellular environment, typically by more than our 10–40 μM (i.e. at least double the IC_50_) estimate [41]. We were constrained to a maximum fura2 dose of 5 μM in the ATPase assay as DMSO also inhibits acto-myosin ATPase at concentrations >0.1%. The maximum concentration of dye we could dissolve in 100% DMSO was 5 mM. Therefore, the real intracellular effects of these dyes may be more pronounced than the 15–25% rates of inhibition captured in vitro (Fig. 4A-B).

These observations have extended implications as Ca^2+^ measurements are a common technique in research, and cardiac Ca^2+^ handling is a central aspect of toxicology testing in industry [28]. When studying drugs known to modulate cardiomyocyte contractility and Ca^2+^ handling, we identified fura2 masked the effect of the drug on both contractility and Ca^2+^ transients compared to an untreated or R-GECO transduced control. The cultured guinea pig cardiomyocyte may be a more extreme example of this issue (compare the effects of fura2 in Fig. 2 to Fig. 1 and Fig. 3), but the myosin isoforms in this system resemble the human adult most closely. Regardless, the early reports of altered contraction and Ca^2+^ seen in the presence of BAPTA-derived dyes, but not protein indicators [43], combined with the work presented here reveals a significant vulnerability for the cardiovascular research community when interpreting results using these dyes. There remains a contemporary concern in adverse dye properties which are of increasing relevance for the growing field of myocardial therapeutics.

### 4.2 Study limitations

There are three principal limitations that impact this study. Firstly, the use of many, but not all, model systems. Secondly, technical aspects of the microscopy infrastructure and sample preparation. Third, the limitation of the protein-based indicators. Taking each point in turn:

We find commonly used BAPTA-derived chemical Ca^2+^ dyes cause varied levels of contractile impairment. The magnitude of the effect depends on the indicator and the model system. Variability may extend to other models, for example, freshly isolated adult rat left ventricular cardiomyocytes appear more resistant to contractile impairment with certain dyes. Cagalinec and co-workers used 1 μM Fluo-3 and achieved ∼5.4% fractional shortening in rat ventricular preparations [44], yet canonical fractional shortening of rat cardiomyocytes without dye loading is typically 9–10% [45], suggesting a degree of contractile impairment remains. Spurgeon and co-workers showed incubation time dependent contractile impairment in rat cardiomyocytes following loading of Indo-1 [46]. Similarly, Shintani and co-workers showed that nanometry measurements of rat neonatal cardiomyocytes were influenced by the addition of Fluo-4, where sarcomere shortening, and relaxation times were profoundly elongated [47].

Experimental technique and hardware can affect signal-to-noise assessments beyond the cell model. Improvements in environmental shielding, sample washing, loading conditions and signal detection could lower the dose of chemical Ca^2+^ dyes below the threshold at which contractile impairment is seen. Ca^2+^ transient acquisition noise is greatly influenced by the detection method. Photomultiplier acquisition (Fig. 1, Fig. 2) is very sensitive with a high sampling rate (1000 Hz) and therefore relatively noisy compared to video-rate acquisition (25 Hz) (Fig. 3) which obtains spatial resolution at the expense of temporal resolution smoothing the data curve.

Genetically encoded indicators require time for functional probe expression, which can be problematic in rapidly de-differentiating models like isolated mouse cardiomyocytes. Agents that prolong culture times, such as blebbistatin [48] or cytochalasin D [49], inhibit the canonical function of myosin and actin, impacting contractile assessment accuracy in this study. Myocardial slices [50], which preserve mechanical architecture for several days, offer an alternative preparation and may bridge the gap between transgenic mouse models and genetically encoded reporters for the cardiovascular system.

### 4.3 Experimental guidance for researchers

Researchers measuring Ca^2+^ and contractility should be cautious when choosing a combination of Ca^2+^ indicator and cellular model. Titration of exogenous chemical Ca^2+^ dye concentration and loading time (or adenoviral MOI and subsequent culture duration for genetically encoded sensors) should be done to determine the minimal concentration that optical systems can handle to generate clear Ca^2+^ signals. Electrical pacing of cardiomyocytes can be used to standardize beat frequency and duration for averaging of multiple transients, but photo-bleaching with intensiometric indicators may limit the efficacy of this approach (Fig. S3 averaged 10 beats, without a 10-fold SNR increase).

Changes in contractility should be corroborated by finding similar results in cardiomyocytes that have not been treated with any indicator. If contractility is different in the presence of the Ca^2+^ indicator it is possible the Ca^2+^ transient captures more than just the intended perturbation to the system. For this reason, we prefer genetically encoded Ca^2+^ sensors such as R-GECO or GCamp6 [51] in cardiomyocytes where possible. While the health and safety aspects of working with biological agents cannot be ignored, a major caveat of Ca^2+^ dye (i.e., the obligatory inclusion of a myosin ATPase inhibitor) use can be routinely avoided. Recent publications investigating contractility and Ca^2+^ simultaneously in iPSC derived engineered heart tissue and 2D iPSC cardiomyocyte cultures with genetically encoded probes GCaMP6 and R-GECO indicate that momentum may be shifting towards this [52,53].

To date, the genetically encoded Ca^2+^ indicators successfully used in adult cardiomyocytes have been almost exclusively intensiometric. This prevents measurement of changes in absolute Ca^2+^ concentration which may be altered by drug or disease as these alterations are missed by indicators such as R-GECO and Fluo-4. Ratiometric dyes such as fura2 or indo1 more accurately calibrate Ca^2+^ concentration. Whilst ratiometric genetically encoded sensors have been developed i.e. YC3.6, Ratiometric Pericam [51], REX-GECO, GEM-GECO [21], their use in cardiomyocytes is not yet widely reported. We believe greater adoption of these indicators, represents an important hurdle to accurately determine changes in cardiomyocyte Ca^2+^ transients without impairment of contractility. Improvements in optical sensitivity of next generation microscopy equipment, coupled with improved techniques to culture and mature iPSC cardiomyocytes or maintain higher quality isolated adult cells in culture could further help achieve this goal.

## Author contributions

PR and MJD conceptualized the study. PR, CR, MJD, AJS, CT, YP designed and interpreted the experiments. PR and AJS, YP performed the chemical dye experiments. PR and AJS made recombinant R-GECO and thin filament proteins. PR, AJS, MAG performed the characterisation of the GECO's. CNB, Y-FC, FAB, performed pilot experiments in iPSC cardiomyocytes. XZ, YAA performed impedance assay measurements. JS, isolated mouse cardiomyocytes. PR, AB, Y-JW performed force fibre experiments. PR and AJS performed guinea pig and mouse cardiomyocyte experiments. YP, VS and CT performed iPSC cardiomyocyte culture and SarcTrack assessments. AJS, CT and YP provided analytic tools. AJS, YP, and PR performed all analysis. PR wrote the 1st draft. MJD, CR, PR, AJS, CT, HW edited the manuscript and all authors provided comments on this manuscript.

## Declaration of Competing Interest

No competing interests declared.

## Appendix A Supplementary data

Supplementary materialSupplementary material
