# Supplementary material for: Comparing the effects of chemical Ca2+ dyes and R-GECO on contractility and Ca2+ transients in adult and human iPSC cardiomyocytes
Source: J Mol Cell Cardiol. 2023 Jul;180:44–57. doi: 10.1016/j.yjmcc.2023.04.008 (PMC10659987; doi:10.1016/j.yjmcc.2023.04.008)
Supplement: Supplementary file 1 [file mmc1.pdf]

Supplemental Figures For:

Comparing the effects of chemical Ca^{2+} dyes and R-GECO on contractility and Ca^{2+} transients in adult and human iPSC cardiomyocytes.

Paul Robinson[1,2*], Alexander J Sparrow[1,2], Yiangos Psaras[1,2], Violetta Steeples[1,2], Jillian Simon[1,2], Connor N Broyles[1,2], Yu-Fen Chang[1,2#], Frances A Brook[1,2], Ying-Jie Wang[1,2], Andrew Blease[1,2], Xiaoyu Zhang[3], Yama A Abassi[3], Michael A Geeves[4], Christopher N Toepfer[1,2,5], Hugh Watkins[1,2,6], Charles Redwood[1,2], Matthew J Daniels[1,2,6,7 *]

[1] *Division of Cardiovascular Medicine, Radcliffe Department of Medicine, University of Oxford, Oxford, UK*
[2] *BHF Centre of Research Excellence, University of Oxford, Oxford, UK*
[3] *Agilent Biosciences, Inc., San Diego, CA 92121, USA*
[4] *Department of Biosciences, University of Kent, Canterbury, UK*
[5] *Department of Genetics, Harvard Medical School, Boston, MA, USA*
[6] *Department of Cardiology, Oxford University NHS Hospitals Trust, Oxford, UK*
[7] *Department of Cardiovascular Sciences, University of Manchester, Manchester, UK*
[#] *Current address: Lumistar Biotechnology Inc., Zebulon Business Center 2F, No.467, Sec. 6, Zhongxiao E. Rd., Nangang Dist., Taipei City 115, Taiwan*
[*]*Joint Corresponding author*
E-mail: paul.robinson@cardiov.ox.ac.uk; matthewdaniels@doctors.org.uk

Contents:

- **Supplemental Methods**

- **Fig. S1.** Commonly used Ca^{2+} and Na^{+} indicators are derived from BAPTA.

- **Fig. S2.** Chemical Ca^{2+} dyes lengthen basal sarcomere length and relaxation time to different degrees in a model dependent manner.

- **Fig. S3.** Transient averaging does not improve signal to noise in a linear relationship.

- **Fig. S4.** Fura2 free salt directly reduces actomyosin ATPase activity.

- **Fig. S5.** Fura2-AM-ester accumulates in adult cardiomyocytes upon normal loading at intracellular concentrations significantly higher than adenovirally expressed R-GECO.

- **Fig. S6.** Unloaded sarcomere shortening curves and extracted parameters to compare RGECO to uninfected and GFP control transduced guinea pig left ventricular cardiomyocytes.

- **Fig. S7.** The green chemical dye Fluo-4 reduces the fluorescence of R-GECO in a dose dependant manner during pairwise multicolour imaging.

- **Fig. S8.** Extracted parameters from unloaded sarcomere shortening curves upon the

application of 250 nM mavacamten.

- **Fig. S9.** Extracted parameters from unloaded sarcomere shortening curves upon the application of 10 µM levosimendan.

- **Fig. S10.** Extracted parameters from unloaded sarcomere shortening curves upon the application of 0.5 µM flecainide.

- **Fig. S11.** Kinetic determination of R-GECO k_{on} and k_{off} by stopped flow.

- **Table. S1.** Extracted parameters from Ca^{2+} transient measurements.

- **Fig. S12.** Contractility/Ca^{2+} loops of guinea pig cardiomyocytes loaded with fura2 or R-GECO in the presence of vehicle or drug (mavacamten, levosimendan or flecainide).

- **Fig. S13.** The Ca^{2+} dye FLIPR Calcium 5 reduces the contractile activity of hiPSC derived cardiomyocytes.

Supplemental Material Online: Methods

Adenoviral Virus design and production

Recombinant adenovirus expressing R-GECO was generated, purified and titre estimated by Welgen Inc. R-GECO viral number estimate is $1x10^{12}$/ml particles count and 5×10^{10} plaque forming units per ml.

Isolation of guinea pig and mouse left ventricular cardiomyocytes.

This investigation was approved by the Animal Welfare and Ethical Review Board at the University of Oxford and conforms to the UK Animals (Scientific Procedures) Act, 1986, incorporating Directive 2010/63/EU of the European Parliament. Guinea pig left ventricular cardiomyocytes were isolated from 400 g male guinea pigs (B&K Grimsdon UK) by collagenase (Worthington Biochemical Corporation, Lakewood, NJ, USA) perfusion via Langendorff apparatus as previously described [1]. Cells were resuspended and plated at ~$1x10^5$ per ml in $ACCITT_3$ media [1, 2]. Recombinant adenovirus was immediately added to 0.75 ml of cell suspension to an estimated multiplicity of infection (MOI) of 444, infected cells were placed at 37°C in a 5% CO_2 atmosphere for 48 hours. This was sufficient to infect 100% of all viable rod-shaped cardiomyocytes with R-GECO.

Mouse left ventricular cardiomyocytes were isolated from 25-30g wild type C57Bl6 mice using a similar method of coronary artery collagenase perfusion as previously described [3, 4]. Cardiomyocytes were kept in storage solution containing 120 mM NaCl, 5.6 mM KCl, 5.0 mM $MgSO_4$ 5 mM sodium pyruvate, 10 mM HEPES pH 7.4, treated with chemical dye and measured for function within 5 hours of isolation.

Stem cell derived cardiomyocytes.

Wild-type hiPSCs with a titin N-terminal enhanced green fluorescent protein (eGFP) tag [5] were kept in culture to passage 35 before differentiation. For passaging, cells were rinsed with phosphate buffered saline (PBS)(Thermo Fisher Scientific) and dissociated with ReLeSR (STEMCELL technologies); this was neutralised with 10 µM Y27632 (Tocris Bioscience) in mTeSR media (STEMCELL Technologies). hiPSCs were passaged in a 1:3 split in 6-well plates pre-coated with Geltrex (Thermo Fisher Scientific). mTeSR media changes were performed at 24 hours with 10 µM Y27632 and henceforth in the absence of Y27632 every 48 hours until cells reached 80% confluence.

Monolayer differentiation was performed via Wnt pathway modulation with small molecule inhibitors, with a protocol modified from Lian *et al* [6]. Briefly, hiPSCs were plated in 6-well plates. Once stem cell confluence reached 80-90%, cells were induced to the mesodermal layer with 12 µM CHIR99021 in RPMI1640 / B27 minus Insulin for 24 hours. This was considered to be day 0 of differentiation. At the end of 24 hours, media were replaced with RPMI1640 / B27 minus insulin. On day 3, cells were induced to cardiac lineage specification with 5 µM IWP2 in RPMI1640 / B27 minus insulin for 48 hours. Cells were then cultured in RPMI1640 / B27 minus insulin until day 7, when insulin was included in the media. Spontaneous contraction was observed on day 9-11 of differentiation. To enhance the purity of cardiomyocytes, cells were subjected to metabolic selection for 48 hours starting on day 11, with glucose-free RPMI1640 / B27 plus insulin. Subsequently, cells were cultured to day 40 in RPMI1640 / B27 plus insulin.

Protein purification.

Recombinant R-GECO was produced by molecular cloning into the pMW172 vector using flanking 5' NdeI and 3' EcoRI restriction sites and an internal HindIII restriction site used as a linker (NEB, USA). R-GECO was expressed for 4 hours in BL21-DE3-pLysS *E.coli* after induction with 0.4 mM Isopropyl β-D-1-thiogalactopyranoside (IPTG). Recovered bacterial pellets were lysed in a buffer containing 25 mM Tris HCl, pH 7.5, 20% sucrose, 1 mM EDTA, 200 mM NaCl, 5 M urea, 0.1% Triton X-

100, and sonicated 3 X 30 seconds. Protein was purified using HiTrap FF chromatography columns and an AKTA-UPC900 FPLC (GE Healthcare, Amersham). R-GECO was purified using sequential anion (pH8.0) exchange chromatography, ammonium sulphate fractionation (to 50%) and finally Hydrophobic Interaction Chromatography (HIC) (in buffer containing 50% ammonium sulphate, 200 mM NaCl 1 mM dithiothretol and 30 mM 4-(2-hydroxyethyl)-1-piperazineethanesulphonic acid (HEPES)). Ion exchange columns were eluted using a gradient of 0-2 M NaCl, the HIC column was eluted with a gradient of 50–0% ammonium sulphate. All eluted protein fractions run on using 12% SDS PAGE gels, stained with Coomassie brilliant blue to assess for purity. Wild type human recombinant TnT, TnI, TnC and Ala-Ser-α-TM[7] were purified as previously described [8]. Troponin complex was reconstituted by dialysis into buffer containing 10 mM imidazole pH 7.0, 1 mM DTT, 0.01% azide, 0.1 mM $CaCl_2$, 6 M urea and 1 M KCl, firstly urea was reduced stepwise from 2 then to 0 M then KCl was reduced stepwise from 1 M to 800 – 600 – 400 – 200 mM KCl in a series of 3 hour dialyses. Tn complex was purified using size exclusion chromatography in 200 mM KCl dialysis buffer, purity was analysed by SDS PAGE. Finally, purified troponin complexes, and are dialysed into buffer containing 5 mM 1,4-Piperazinediethanesulphate pH 7.0, 3.87 mM $MgCl_2$, 1 mM DTT for ATPase assay experiments. Actin was extracted from rabbit skeletal muscle as previously described [9, 10]. Bovine cardiac myosin-S1 was extracted and purified from whole heart tissue using a method adapted from Siemankowski and White [11]. Briefly; 70 g of left ventricular tissue was homogenised in 400 ml of skinning solution containing 25 mM Na_2EGTA, 50 mM MOPS pH 6.8, 6 mM $MgCl_2$, 6 mM Na_2ATP, 2 mM DTT, 50 µg/ml Tosyl-L-lysyl-chloromethane hydrochloride (TLCK), 10 µg/ml Tosyl-L-Arginine Methyl Ester (TAME) and 0.2 mM PMSF at pH 6.8 at 4 ^{0}C and stirred gently for 30 minutes. The solution was centrifuged at 12,000 xg for 15 minutes. The pellet was resuspended in 600 ml of extraction buffer containing 300 mM KCl, 10 mM $Na_4P_2O_7$, 1 mM $MgCl_2$, 5 mM K_2EGTA, 150 mM KH_2PO_4, 2 mM DTT, 50 µg/ml TLCK, 10 µg/ml TAME and 0.2 mM PMSF at pH 6.8). Extraction was performed on ice over 90 minutes with gentle stirring and then spun for 10 minutes at 12,000 xg. The supernatant volume was measured and diluted with 20 volumes of chilled low salt solution containing, 1 mM DTT and 2 mM

$MgCl_2$ 10 mM MOPS pH6.8 and then left on ice for two hours and subsequently centrifuged at 20,000 xg for 20 minutes. The pellet was re-suspended in 200 ml of acto-myosin dissolving solution containing 800 mM KCl, 50 mM MOPS pH 6.8, 5 mM $MgCl_2$, 2 mM 2-mercaptoethanol at pH 6.8. The ionic strength of the solution was then reduced by the addition of diluting solution containing 50 mM MOPS pH 6.8, 5 mM $MgCl_2$ and 20 mM Na_2ATP at pH 6.8 to give a final ionic strength of 310 mM. The solution was then centrifuged at 75000 xg for 2 hours, the supernatant was measured and diluted by 8 volumes of low salt buffer and left on ice for 2 hours. The solution was then spun at 20,000 xg for 10 minutes and the pellet was dissolved in 150 ml of high salt solution containing 500 mM NaCl, 2 mM $MgCl_2$, 5 mM 2-mercaptoethanol and 80 mM MOPS pH 6.8. Whole myosin solution was stored at -20 ^{o}C in 50% glycerol. Chymotryptic digest of myosin to purify soluble S1 catalytic heads was performed by dialysing 100 ml of glycerol myosin stock against buffer containing, 75 mM NaCl, 50 mM NaH_2PO_4 / Na_2HPO_4 pH 6.8, 50mM MOPS pH6.8, 1mM DTT. TLCK treated chymotrypsin from bovine pancreas was added to a final concentration of 50 µg / ml and stirred overnight at 4 ^{o}C. The digest was stopped with the addition of 1 mM PMSF and immediate dialysis against buffer containing 50 mM MOPS pH 6.8 and 1 mM DTT. Centrifugation at 20000 xg for 10 minutes removed any insoluble myosin rod contaminant and soluble myosin S1 was purified using a 5 ml QFF anion exchange column. The protein was stored in 50 % glycerol at – 80 C.

In vitro acto-myosin activated myosin ATPase assays.

ATPase assays were undertaken using 3.5 µM actin, 0.5 µM myosin S1, 0.5 µM Ala-Ser-α-TM and 0.5 µM Tn complex in buffer described above [8, 12]. To remove unbound troponin each stock was centrifuged at 384000 x*g* for 13 minutes. Supernatants were discarded, actin pellets were recovered in buffer containing 5 mM 1,4-Piperazinediethanesulphate pH 7.0, 3.87 mM $MgCl_2$, 1 mM DTT to an equal volume of the discarded supernatant. Reaction mixtures were aliquoted and set to a range of free Ca^{2+} concentrations between 3.16 nM (pCa 8.5) and 31.6 µM (pCa 4.5) using 1 mM EGTA and the appropriate corresponding concentration of $CaCl_2$, calculated using Maxchelator software

(http://maxchelator.stanford.edu/CaEGTA-TS.htm). Calcium-sensitivity data was fitted to the Hill equation using Kaleidagraph (Synergy Software, Inc, USA).

Hill equation: $$A = A_{min} + \left[\frac{(A_{max} - A_{min})}{1+10^{(pCa-pCa_{50})\times n_H}}\right]$$

Where: A = ATPase rate; A_{min} = Minimum ATPase rate; A_{max} = Maximum ATPase rate; pCa = -log [Ca^{2+}]; pCa_{50} = - log [Ca^{2+}] required for half maximum ATPase activity; n_H = Hill coefficient.

Cardiac fibre force measurements.

Cardiac muscle fibres were dissected from wild type C57Bl6 mice at 10 weeks old. Fibres were chemically demembranated overnight in solution containing 10 mM EGTA, 5.6 mM $MgCl_2$, 100 mM KCl, 20 mM Imidazole, 5 mM ATP, 10 mM Creatine phosphate, 50% glycerol, 1 % triton X100 pH 7.0. Force was measured using microscope mountable 1400A Permeabilized Fibre System from Aurora Scientific as previously described [13]. Briefly, the T clipped fibre measuring approximately 3 mm by 0.1 mm was attached to the length controller and force transducer and allowed to equilibrate in a low Ca^{2+} (pCa 9.0) solution containing. The fibre was stretched to a sarcomere length of 2.2 nm, measured by laser diffraction and the attached graticule provided with the apparatus. The fibre was pre-stretched in a pCa4.0 solution for 10 minutes and returned to the relaxing solution at pCa9 where sarcomere length was recalibrated, and fibre dimensions were determined using the microscope graticule under 10x magnification. The maximally activated force was then measured with a pCa4 solution containing 0.2% DMSO for 5-10 minutes until the force plateau was achieved. The fibre was then moved to pCa 4 solution containing sequentially increasing fura2 concentrations (0.2, 1.0 and 5.0 µM) for 5-10 minutes, DMSO concentration was maintained at 0.2% in each solution. Finally the fibre was returned to the pCa4.0 DMSO solution to correct for rundown of force throughout the experiment. Four fibres were run in this way with a further 3 fibres moving sequentially from 5.0, 1.0 and 0.2 µM of fura2 in a reversal of treatment sequence to ensure that the fibre was not affected from solution-specific degradation in force. Corrected raw force measurements were normalised to the

cross-sectional area of the fibre assuming an elliptical shape to give the final area calculated from width and depth measurements.

Calcium binding, K_d and kinetic calculations.

To measure steady state Ca^{2+} binding affinity (K_d) for R-GECO 3 µM of protein was dialysed into buffer containing 130 mM NaCl, 10 mM HEPES, 1mM dithiothretol, pH 7.3. A range of free [Ca^{2+}] was set between 3.16 nM (pCa 8.5) and 31.6 µM (pCa 4.5) using 1 mM EGTA and the appropriate corresponding concentration of $CaCl_2$. Steady state fluorescence readings were made using a 544 nm excitation filter and 590/10 nm emission filter, in an Ultraclear bottom 96 well microplate using a FLUOstar Omega plate reader (BMG LABTEC, Ortenberg, Germany). Resultant fluorescence emission intensities were plotted vs free [Ca^{2+}] and fitted to the Hill equation to calculate K_d values for each protein and temperature (25 and 37 °C) and [Mg^{2+}] (0, 1.3 and 3.87 µM).

To measure Ca^{2+} displacement (k_{off}) of 125 nM R-GECO; 5 µM $CaCl_2$ was mixed with 5 mM EGTA in a buffer containing 130 mM NaCl, 10 mM HEPES 1.3 mM $MgCl_2$, 1 mM dithiothretol, pH 7.3 with NaOH. R-GECO was loaded into a Stopped-flow system (HiTech Scientific, Bradford-on-Avon, UK), concentrations after mixing 1:1 in the stopped-flow. Fluorescence was excited at 546 nm (100W Xe/Hg lamp and monochromator) and emission measured through a OG-590 glass filter. Resultant data was fitted to a single exponential decrease in fluorescence of 50-100%, which was dependent on temperature. To measure calcium binding (k_{on}), 125 nM R-GECO was measured at 10 µM free calcium by mixing with a buffer containing 2.125 mM Ca.EGTA and 0.2 mM EGTA, using an identical fluorescence filter setup in the stopped flow apparatus as above. A large fluorescence increase was detected upon addtion of Ca^{2+} was fitted to a single exponetial. The observed single exponential rate constant, k_{obs}, was extracted for k_{on} and k_{off}, between 25 and 37°C at ~2.5°C intervals. To estimate the dissociation constant we used the equations $K_d = k_{obs\text{-}}$off [Ca]/ k_{obs} on. Of note, calculated the K_d value was analogous to those obtained the in previous steady state experiment.

Measurement of sarcomere shortening and Ca^{2+} transients in adult cardiomyocytes.

Sarcomere shortening and Ca^{2+} transient measurements were performed using IonOptix µstep apparatus and the manufacturers' standard operating instructions as previously described [1]. Briefly: cultured cardiomyocytes were treated with fura2 Ca^{2+} indicator by incubation with either 0.2, 1.0 or 5.0 µM fura2-AM ester, Fluo-4-AM ester, Rhod-2-AM ester or SBFI-AM ester (Life Technologies) in the presence of 2 µM F127 Pluronic in 'perfusion buffer' (150 mM NaCl, 10 mM HEPES, 7 mM glucose, 1 mM $MgCl_2$, 1 mM KCl, 0.3 mM NaH_2PO_3, pH 7.4 with NaOH) containing 250 µM $CaCl_2$ for 5 minutes, followed by a 10 minute wash in perfusion buffer containing 500 µM $CaCl_2$ to remove any excess label. For experiments using SBFI 0.2, 1.0 or 5.0 µM were treated in the same manner. Experiments for guinea pig cardiomyocytes were carried out at 1.8 mM Ca^{2+} and a pacing frequency of 0.5 Hz, whilst mouse cardiomyocytes used 1.4 mM $CaCl_2$ and 3 Hz pacing. All contracting cardiomyocytes were measured for contractility and fura2 / Fluo-4 Ca^{2+} (Rhod-2 fluorescence was unable to be measured due to the limitations of the IonOptix photomultiplier system), any cells displaying asynchronous contractility, excessive blebbing/dysmorphology were ignored for acquisition. To compare sarcomere shortening and fura2 Ca^{2+} transients in the presence of contractility or Ca^{2+} modifying small molecules mavacamten (250 nM), levosimendan (10 µM) and flecainide (0.5 µM), 200 µl of cultured (48 Hours) cardiomyocytes were incubated in either drug or DMSO vehicle for 5 minutes. The entire cell preparation was pipetted onto the microscope perfusion chamber buffer containing the either drug or vehicle was perfused at 0.75 ml/minute for the duration of the experiment. At least 3 cell preparations were analyzed for each DMSO/drug comparison. No differences or clustering was observed between each cell preparation assessed.

Fluorescent Ca^{2+} transient imaging of adult cardiomyocytes by video capture.

For R-GECO Ca^{2+} transients in guinea pig cardiomyocytes were acquired using an Olympus IX81 inverted microscope (Olympus, Japan) with a C-9100-13 EMCCD camera (Hamamatsu, Japan). Videos

of 0.5 Hz electrically paced cardiomyocytes at 37°C were measured at 25 fps (560/25 nm excitation, 620/60 nm emission with a 565 nm dichroic mirror). Ca^{2+} transients from Fluo-4 and Rhod-2 indicators were acquired through an Olympus UPlanFLN 10x lens (NA 0.3), using GFP (525/50 nm (emission), 485/20-25 nm (excitation)) and RFP filters. R-GECO transduced guinea pig cardiomyocytes were treated with 250 nM mavacamten, 10 µM levosimendan, or 0.5 µM flecainide for 20 minutes. Raw image data was extracted using xcellence rt (Olympus), and analysed in Excel (Microsoft) as previously described [14].

hiPSC derived cardiomyocyte contraction analysis with SarcTrack.

Cardiomyocytes were split into six groups: DMSO, fura2, Rhod-2, Fluo-4, SBFI and red genetically encoded Ca^{2+} indicator for optical imaging (R-GECO). All chemical indicators were used at 0.2, 1 and 5 µM in tyrode-HEPES buffer (150 mM NaCl, 5 mM HEPES, 7 mM glucose, 1.8 mM KCl, 350 µM NaH_2PO_3 and 420 µM $CaCl_2$, pH 7.4). These were treated in low calcium (200 µM) tyrode-HEPES buffer for 5 minutes, followed by three 5-minute sequential washes with low, medium (300 µM) and high (420 µM) calcium buffer; all buffers were warmed to 37°C. Cardiomyocytes were adenovirally transduced for 24 hours with R-GECO 24 hours post seeding, as previously described [15] at multiplicity of infection =150; controls were provided by non-transduced cardiomyocytes.

Imaging was performed with an Olympus IX81 inverted microscope (Olympus, Japan) with an Andor iXon Ultra 897 EMCCD camera (Oxford Instruments, UK). Cell videos were captured at 37°C at 50 frames per second under electrical pacing at 1 Hz, at 485 / 20-25 nm excitation and 525 / 50 emission with at 495 nm dichroic mirror. Videos were processed and parameters extracted with SarcTrack [16].

Ca^{2+} transient signal-to-noise acquisition and analysis.

Ca^{2+} imaging was performed for fura2 and Fluo-4 in adult cardiomyocytes by photomultiplier acquisition using and IonOptix µStep system. Limitations on photo multiplier sensitivity to light wave lengths above 550 nm prevented acquisition of Rhod-2 and R-GECO using these optics. Ca^{2+} dependent fluorescence signals were also acquired for hiPSC derived cardiomyocytes already mounted for sarcomere imaging, and under the same conditions. Recordings were only performed for Rhod-2, Fluo-4 and R-GECO due to limitations in microscopy equipment. Resultant raw traces were extracted was performed by IonWizard (IonOptix) or with CalTrack [15]. The signal to noise ratio was calculated by dividing the peak trace amplitude with the standard deviation of all baseline signal.

Intracellular concentration estimation of fura2 and R-GECO in adult guinea pig left ventricular cardiomyocytes.

Average cardiomyocyte cell volume was estimated in a method adapted from Jiang et al as previously described [17], Briefly, Freshly isolated adult guinea pig left ventricular cardiomyocytes were attached to poly-D-lysine coated slides using a StatSpin cytofuge at 700 rpm for 2 min and fixed using 4 % paraformaldehyde. Phase contrast z stacked images were acquired by confocal microscopy, average cell length width and depth were calibrated using an internal graticule on a Leica TCS SP5 X confocal microscope.

To generate standard calibration curves for each fluorophore tested, fura2 free salt was diluted 3, 10 and 30 nM and recombinant R-GECO was diluted to 0.5, 1 and 5 nM in buffer containing 20 mM HEPES pH7.0, 120 mM NaCl, 1 mM $MgCl_2$ and 1 mM EGTA. The fluorescence emission at 510 nm at with an excitation of 340 nm was measured for fura2, whilst R-GECO was measured at an emission and excitation wavelength of 605 nm and 565 nm respectively using a RF-1501 spectrofluorimeter (Shimadzu, Japan).

To estimate intracellular concentration of each fluorophore 1x10^5 rod shaped cardiomyocytes (estimated using a 0.1 mm haemocytometer) were treated with 0.2, 1 and 5 µM of fura2 as described above. For R-GECO 3x10^5 cells were transduced with recombinant adenovirus expressing R-GECO at and MOI of 1000, 200 or 40 and cultured for 48 hours in $ACCITT_3$ media, 1X10^5 rod shaped cells were taken for lysis. Cells were washed in fluorescence buffed above twice to remove any residual Ca^{2+} from the loading or culture media, they were then lysed in 0.5 ml buffer containing 3 % triton-X100, 20 mM HEPES pH7.0, 120 mM NaCl, 1 mM $MgCl_2$ and 1 mM EGTA for 10 minutes, cell debris was removed by centrifugation at 17000 xg for 10 minutes, supernatant was then used to estimate the fluorescent unit yield. The concentration of intracellular fluorophore concentration was calculated using the equation:

$$[\mathrm{F}] = \mathrm{F}_{\mathrm{sample}} * \left[\frac{(\mathrm{Cn} - \mathrm{Cv})}{\mathrm{Bv}}\right]$$

Where: F_{sample} = the fluorescence yield of the sample, *Cn* = cell number, *Cv* = cell volume, *Bv* = buffer volume.

Finally, intracellular concentration was determined for each loading concentration of fura2 or MOI of R-GECO, by taking the volume adjusted fluorescence calculation and applying the respective linear regression calculation determined from the calibration plots acquired above.

Impedance assessment of contractility.

iCell cardiomyocytes (CDI) were seeded at 20,000 cells/well into RTCA plates and loaded onto an xCELLigence RTCA Cardio system (ACEA bioscience) housed in a humidified incubator at 37°C with a 5% CO_2 atmosphere, this was interrupted for media changes on alternate days. Contractility was monitored via impedance assessment at 12.9 millisecond intervals. At day 14 post seeding, with robust signals apparent in all wells, cells were treated with FLIPR 5 (Molecular Devices) around the recommended 1x final working concentration. The recommended loading phase for FLIPR Calcium 5 is 1 hour, recording continued for 24 hours. Raw transient data for the windows indicated together

with transient amplitude and beat rate over the first two hours of the experiment were extracted using the RTCA software and prepared for presentation using Microsoft Excel.

Statistics.

Each cell preparation (Guinea pig, mouse or hiPSC cardiomyocytes) was split into 13 groups: loaded with 0.2, 1.0, 5.0 µM fura2, Fluo-4, Rhod-2 or SBFI AM esters and compared to a DMSO incubated control. R-GECO comparisons in guinea pig cardiomyocytes and hiPSC cardiomyocytes were between uninfected (non DMSO treated) controls. Drug treatments compared pre and post treatment for each drug for uninfected, fura2 treated or R-GECO infected groups. For all cell experiments, at least 3 separate preparations were used, n gives the total cell number for each analysis, no evidence of clustering was observed for all data sets analysed. ATPase analysis was compared from 5 separate independent measurements and at least 2 thin filament reconstitutions. All data was assessed for normality using a D'Agostino & Pearson test and, where appropriate, were compared using students t-test, or non-parametric Mann-Whitney test for two data sets or One-way ANOVA or non-parametric Kruskal-Wallis test with Tukey post hoc analysis for multiple comparisons where multiple data sets are compared to a single control.

1. Robinson P, Liu X, Sparrow A, Patel S, Zhang YH, Casadei B, Watkins H, Redwood C. Hypertrophic cardiomyopathy mutations increase myofilament Ca(2+) buffering, alter intracellular Ca(2+) handling, and stimulate Ca(2+)-dependent signaling. *J Biol Chem* 2018;**293**:10487-10499.
2. Ellingsen O, Davidoff AJ, Prasad SK, Berger HJ, Springhorn JP, Marsh JD, Kelly RA, Smith TW. Adult rat ventricular myocytes cultured in defined medium: phenotype and electromechanical function. *Am J Physiol* 1993;**265**:H747-754.
3. Sears CE, Bryant SM, Ashley EA, Lygate CA, Rakovic S, Wallis HL, Neubauer S, Terrar DA, Casadei B. Cardiac neuronal nitric oxide synthase isoform regulates myocardial contraction and calcium handling. *Circ Res* 2003;**92**:e52-59.
4. Carnicer R, Hale AB, Suffredini S, Liu X, Reilly S, Zhang MH, Surdo NC, Bendall JK, Crabtree MJ, Lim GB, Alp NJ, Channon KM, Casadei B. Cardiomyocyte GTP cyclohydrolase 1 and tetrahydrobiopterin increase NOS1 activity and accelerate myocardial relaxation. *Circ Res* 2012;**111**:718-727.

5. Sharma A, Toepfer CN, Ward T, Wasson L, Agarwal R, Conner DA, Hu JH, Seidman CE. CRISPR/Cas9-Mediated Fluorescent Tagging of Endogenous Proteins in Human Pluripotent Stem Cells. *Curr Protoc Hum Genet* 2018;**96**:21 11 21-21 11 20.
6. Lian X, Hsiao C, Wilson G, Zhu K, Hazeltine LB, Azarin SM, Raval KK, Zhang J, Kamp TJ, Palecek SP. Robust cardiomyocyte differentiation from human pluripotent stem cells via temporal modulation of canonical Wnt signaling. *Proc Natl Acad Sci U S A* 2012;**109**:E1848-1857.
7. Monteiro PB, Lataro RC, Ferro JA, Reinach Fde C. Functional alpha-tropomyosin produced in Escherichia coli. A dipeptide extension can substitute the amino-terminal acetyl group. *J Biol Chem* 1994;**269**:10461-10466.
8. Robinson P, Mirza M, Knott A, Abdulrazzak H, Willott R, Marston S, Watkins H, Redwood C. Alterations in thin filament regulation induced by a human cardiac troponin T mutant that causes dilated cardiomyopathy are distinct from those induced by troponin T mutants that cause hypertrophic cardiomyopathy. *J Biol Chem* 2002;**277**:40710-40716.
9. Pardee JD, Spudich JA. Purification of muscle actin. *Methods Cell Biol* 1982;**24**:271-289.
10. Weeds AG, Taylor RS. Separation of subfragment-1 isoenzymes from rabbit skeletal muscle myosin. *Nature* 1975;**257**:54-56.
11. Siemankowski RF, White HD. Kinetics of the interaction between actin, ADP, and cardiac myosin-S1. *J Biol Chem* 1984;**259**:5045-5053.
12. Robinson P, Griffiths PJ, Watkins H, Redwood CS. Dilated and hypertrophic cardiomyopathy mutations in troponin and alpha-tropomyosin have opposing effects on the calcium affinity of cardiac thin filaments. *Circ Res* 2007;**101**:1266-1273.
13. Smart N, Riegler J, Turtle CW, Lygate CA, McAndrew DJ, Gehmlich K, Dube KN, Price AN, Muthurangu V, Taylor AM, Lythgoe MF, Redwood C, Riley PR. Aberrant developmental titin splicing and dysregulated sarcomere length in Thymosin beta4 knockout mice. *J Mol Cell Cardiol* 2017;**102**:94-107.
14. Sparrow AJ, Sievert K, Patel S, Chang YF, Broyles CN, Brook FA, Watkins H, Geeves MA, Redwood CS, Robinson P, Daniels MJ. Measurement of Myofilament-Localized Calcium Dynamics in Adult Cardiomyocytes and the Effect of Hypertrophic Cardiomyopathy Mutations. *Circ Res* 2019;**124**:1228-1239.
15. Psaras Y, Margara F, Cicconet M, Sparrow AJ, Repetti G, Schmid M, Steeples V, Willcox JA, Bueno-Orovio A, Redwood C, Watkins H, Robinson P, Rodriguez B, Seidman JG, Seidman CE, Toepfer CN. CalTrack: High Throughput Automated Calcium Transient Analysis in Cardiomyocytes. *Circ Res* 2021.
16. Toepfer CN, Sharma A, Cicconet M, Garfinkel AC, Mucke M, Neyazi M, Willcox JAL, Agarwal R, Schmid M, Rao J, Ewoldt J, Pourquie O, Chopra A, Chen CS, Seidman JG, Seidman CE. SarcTrack. *Circ Res* 2019;**124**:1172-1183.
17. Jiang H, Hooper C, Kelly M, Steeples V, Simon JN, Beglov J, Azad AJ, Leinhos L, Bennett P, Ehler E, Kalisch-Smith JI, Sparrow DB, Fischer R, Heilig R, Isackson H, Ehsan M, Patone G, Huebner N, Davies B, Watkins H, Gehmlich K. Functional analysis of a gene-edited mouse model to gain insights into the disease mechanisms of a titin missense variant. *Basic Res Cardiol* 2021;**116**:14.

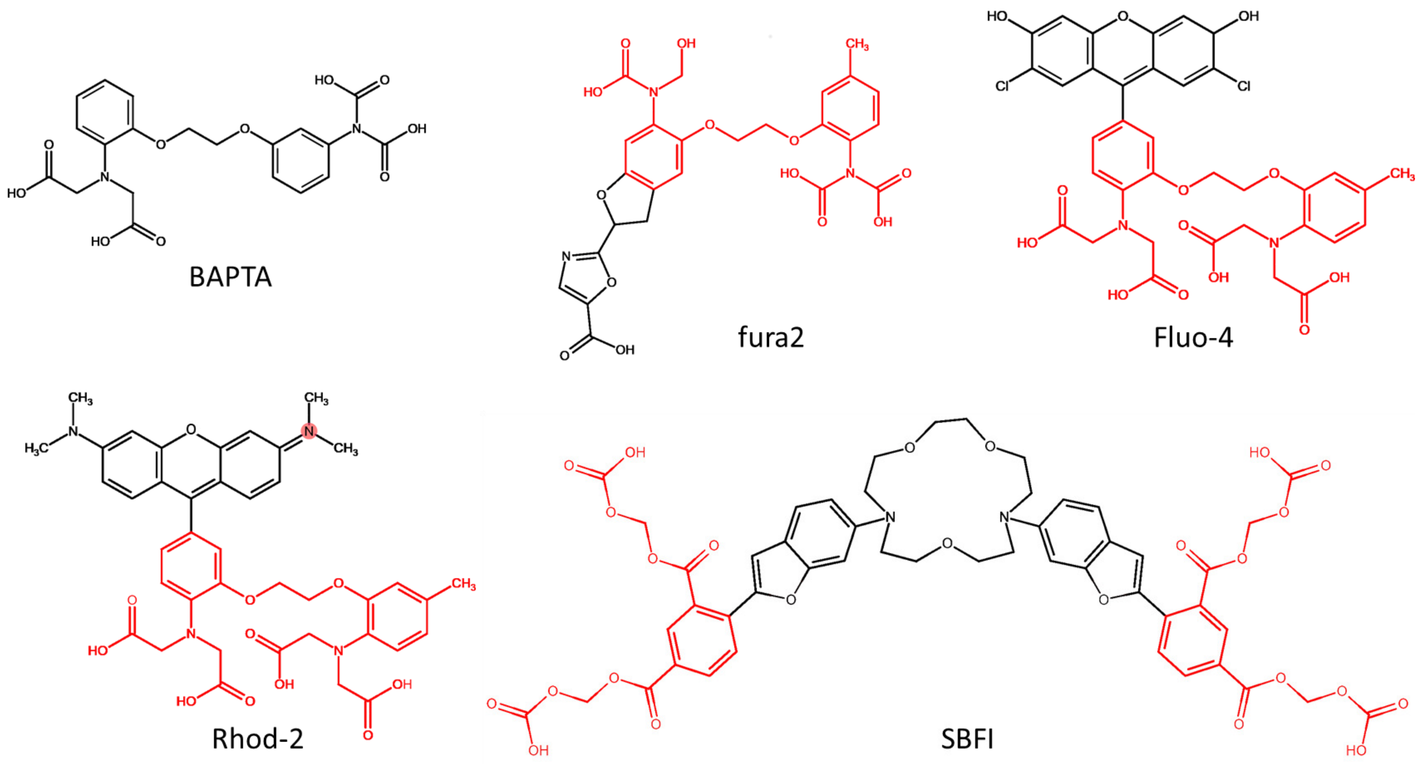

Fig. S1. Commonly used Ca^{2+} and Na^{+} indicators are derived from BAPTA.
Chemical structures show the core BAPTA molecule (red) (K_d = 160nM) in addition to fluorescent reporter side chain which determine fluorescent wavelength emission (EM) and excitation (EX), fua2: EM = 340/380 nm, EX = 510 nm, K_d = 145nM. Fluo-4: : EM = 494 nm, EX = 516 nm, K_d = 345nM. Rhod-2 : EM = 553 nm , EX = 576, K_d = 370nM. SBFI : EM = 340/380, EX = 510, K_d = 3.8 mM.

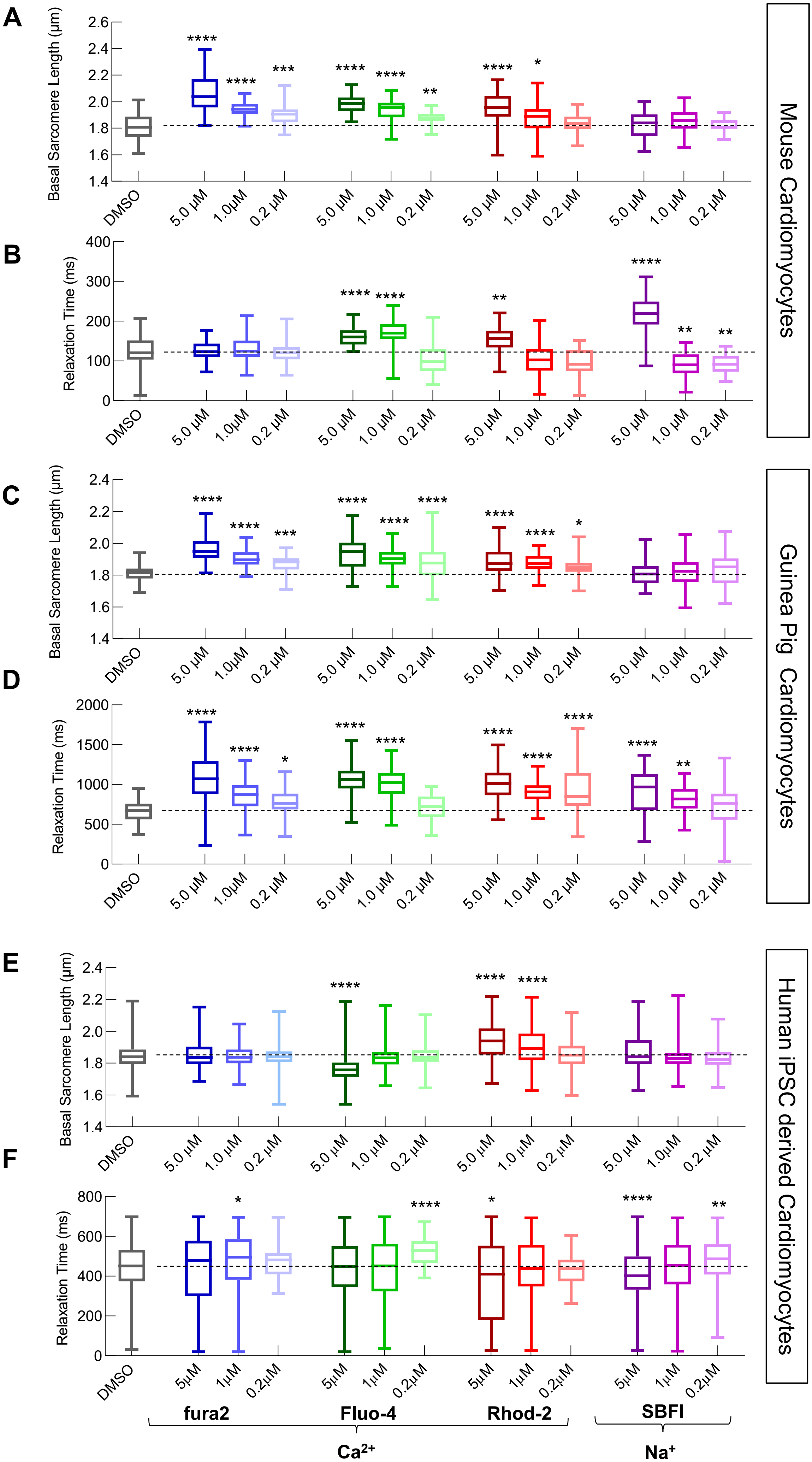

A
Basal Sarcomere Length (µm)
2.6
2.4
2.2
2.0
1.8
1.6
1.4
DMSO
5.0 µM
1.0µM
0.2 µM
5.0 µM
1.0 µM
0.2 µM
5.0 µM
1.0 µM
0.2 µM
5.0 µM
1.0 µM
0.2 µM
****
****
***
****
****
**
****
*
B
Relaxation Time (ms)
400
300
200
100
0
DMSO
5.0 µM
1.0µM
0.2 µM
5.0 µM
1.0 µM
0.2 µM
5.0 µM
1.0 µM
0.2 µM
5.0 µM
1.0 µM
0.2 µM
****
****
**
****
**
**
Mouse Cardiomyocytes
C
Basal Sarcomere Length (µm)
2.4
2.2
2.0
1.8
1.6
1.4
DMSO
5.0 µM
1.0µM
0.2 µM
5.0 µM
1.0 µM
0.2 µM
5.0 µM
1.0 µM
0.2 µM
5.0 µM
1.0 µM
0.2 µM
****
****
***
****
****
****
****
****
*
D
Relaxation Time (ms)
2000
1500
1000
500
0
DMSO
5.0 µM
1.0µM
0.2 µM
5.0 µM
1.0 µM
0.2 µM
5.0 µM
1.0 µM
0.2 µM
5.0 µM
1.0 µM
0.2 µM
****
****
*
****
****
****
****
****
****
**
Guinea Pig Cardiomyocytes
E
Basal Sarcomere Length (µm)
2.4
2.2
2.0
1.8
1.6
1.4
DMSO
5.0 µM
1.0 µM
0.2 µM
5.0 µM
1.0 µM
0.2 µM
5.0 µM
1.0 µM
0.2 µM
5.0 µM
1.0µM
0.2 µM
****
****
****
F
Relaxation Time (ms)
800
600
400
200
0
DMSO
5µM
1µM
0.2µM
5µM
1µM
0.2µM
5µM
1µM
0.2µM
5µM
1µM
0.2µM
*
****
*
****
**
fura2
Fluo-4
Rhod-2
SBFI
Ca2+
Na+
Human iPSC derived Cardiomyocytes

Fig. S2. Chemical Ca^{2+} dyes lengthen basal sarcomere length and relaxation time to different degrees in a model dependent manner.

Averaged single cell relaxation time and basal sarcomere length changes for cells loaded with 0.2 µM, 1.0 µM or 5.0 µM fura2 (Blue); Fluo-4 (green), Rhod-2 (red) and SBFI (purple). Box and whisker plots give the median average, interquartile range (box) and minimum and maximum data spread (whiskers). * = p<0.05 and ** = p<0.01 ,*** = p<0.001 **** = p<0.0001 using non-parametric Kruskal-Wallis test. *n*=37-52 cells from 3 different isolations for mouse cardiomyocytes (A and B), (*n*=47-68 cells from 3 different isolations for guinea pig cardiomyocytes (C and D), *n*=1321-198 cells from 3 separate clonal seedings for iPSC cardiomyocytes (E and F).

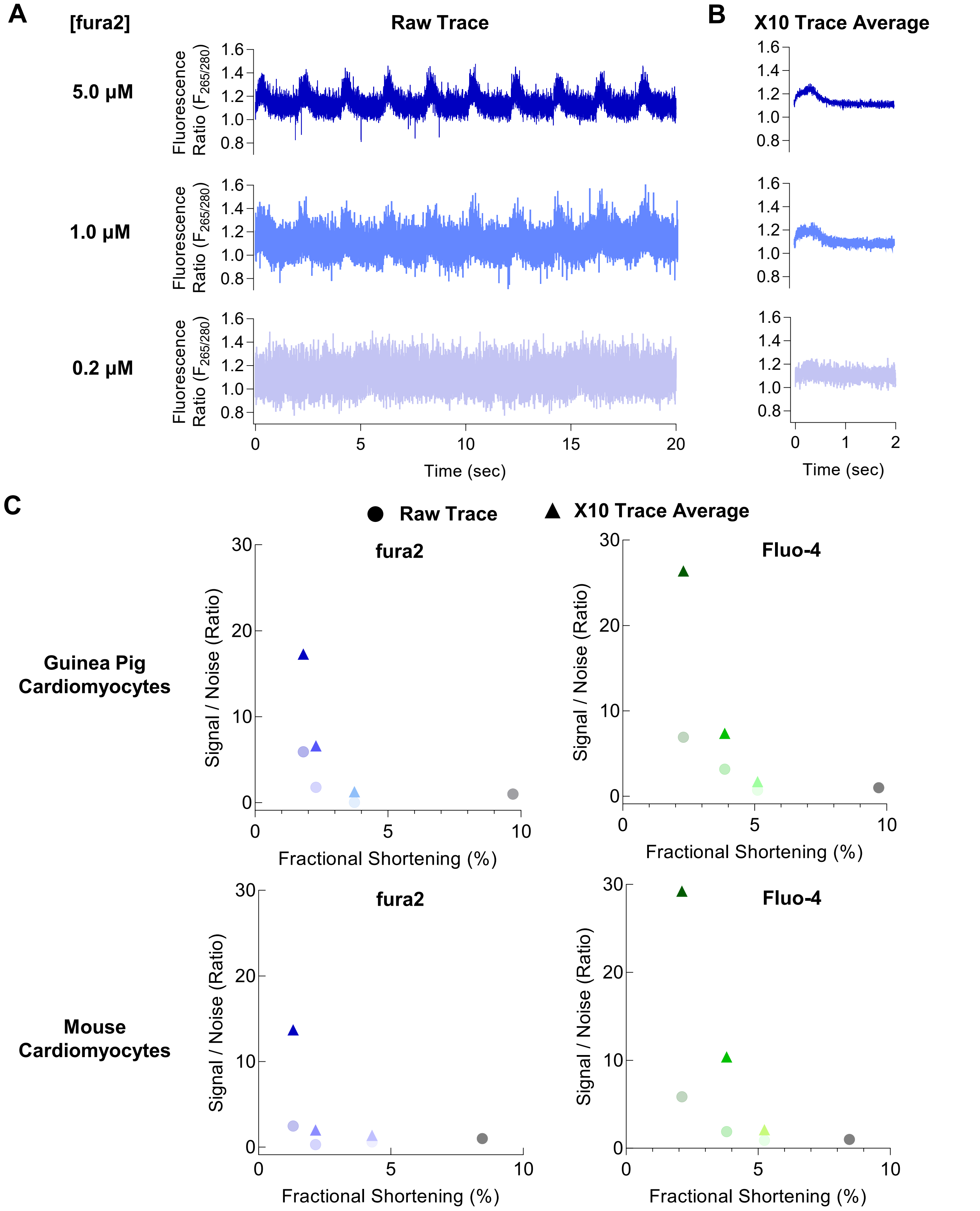

Fig. S3. Transient averaging does not improve signal to noise in a linear relationship. Representative raw Ca^{2+} transient traces for fura2 treated adult guinea pig left ventricular cardiomyoctes, collected via photomultiplier photon acquisition at 1000 Hz, are shown for 0.2. 1.0 and 5.0 μM respectively (A). Averaging the 10 transients shows improvement in the signal to noise of the resultant trace (B). Average signal to noise ratio extracted from the traces are plotted verses fractional shortening for fura2 and Fluo-4 treated guineapig and mouse cardiomyocytes (C). Circles give the average signal to noise of the raw traces (*n*=20 cells from 3 separate isolations) previously given in Fig.1D,F and 2D,F respectively, triangles give the average signal to noise from averages of 10 transients from each raw data set (*n*=15).

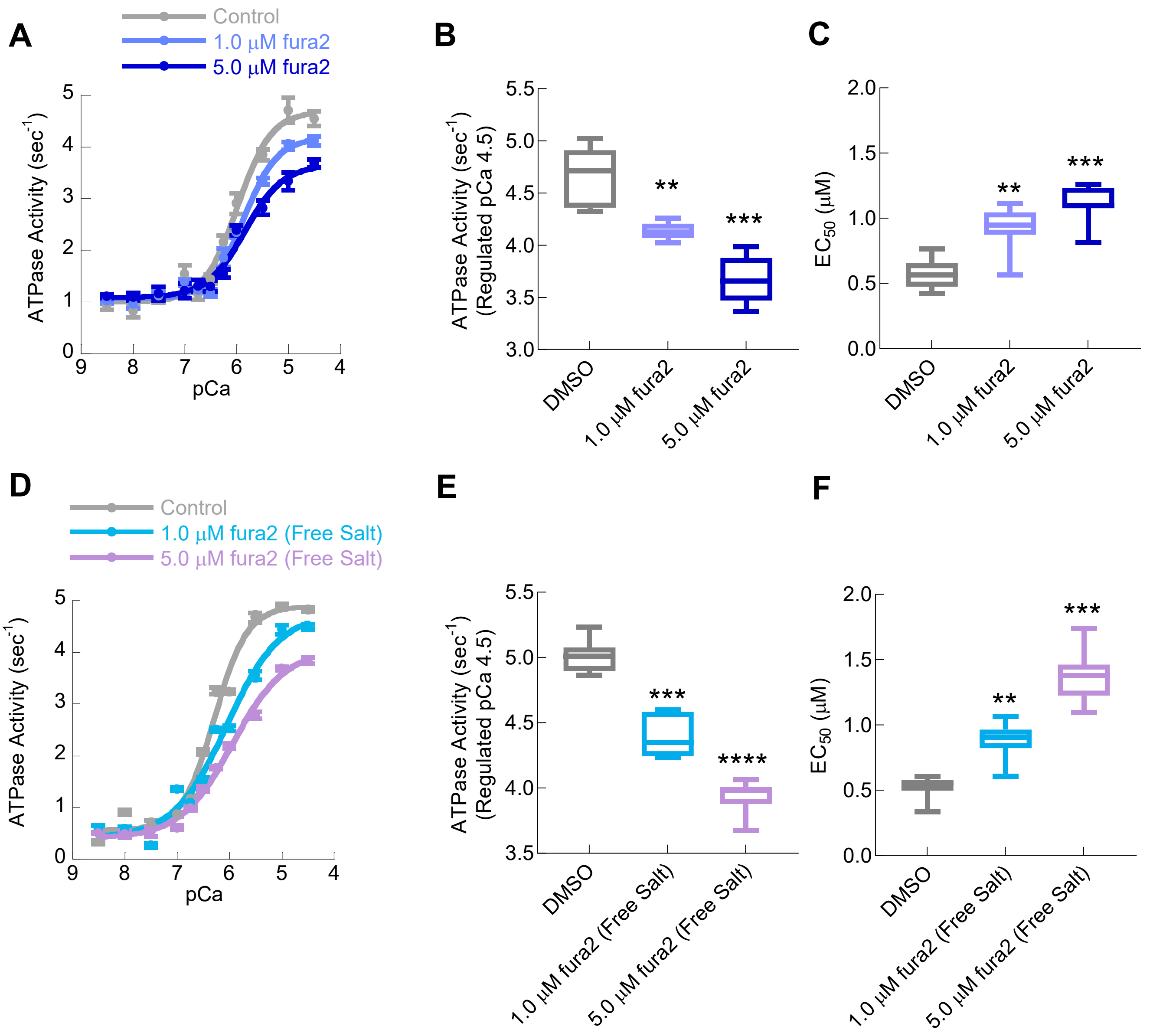

Fig. S4. fura2 free salt directly reduces acto-myosin ATPase activity. The action of fura2 free salt was performed to ensure AM esterification di not interfere with the ATPase assay result from Figure 4. Myofilament function was assessed using *in vitro* actin activated acto-myosin S1 ATPase assays using thin filaments reconstituted with bovine cardiac troponin and human recombinant Ala-Ser-tropomyosin. The ATPase:pCa relationship was determined for reconstituted myofilament preparations in the presence of DMSO (grey), 5.0 µM (blue) or 1.0 µM (purple) fura2. B and C give the extracted parameters for the maximum ATPase activity, and the EC_{50} respectively. Box and whisker plots B,C,D and E give the median average, interquartile range (box) and minimum and maximum data spread (whiskers). * = p<0.05, *** = p<0.001, and **** = p<0.0001 using one way ANOVA, *n*=6 separate assays recordings.

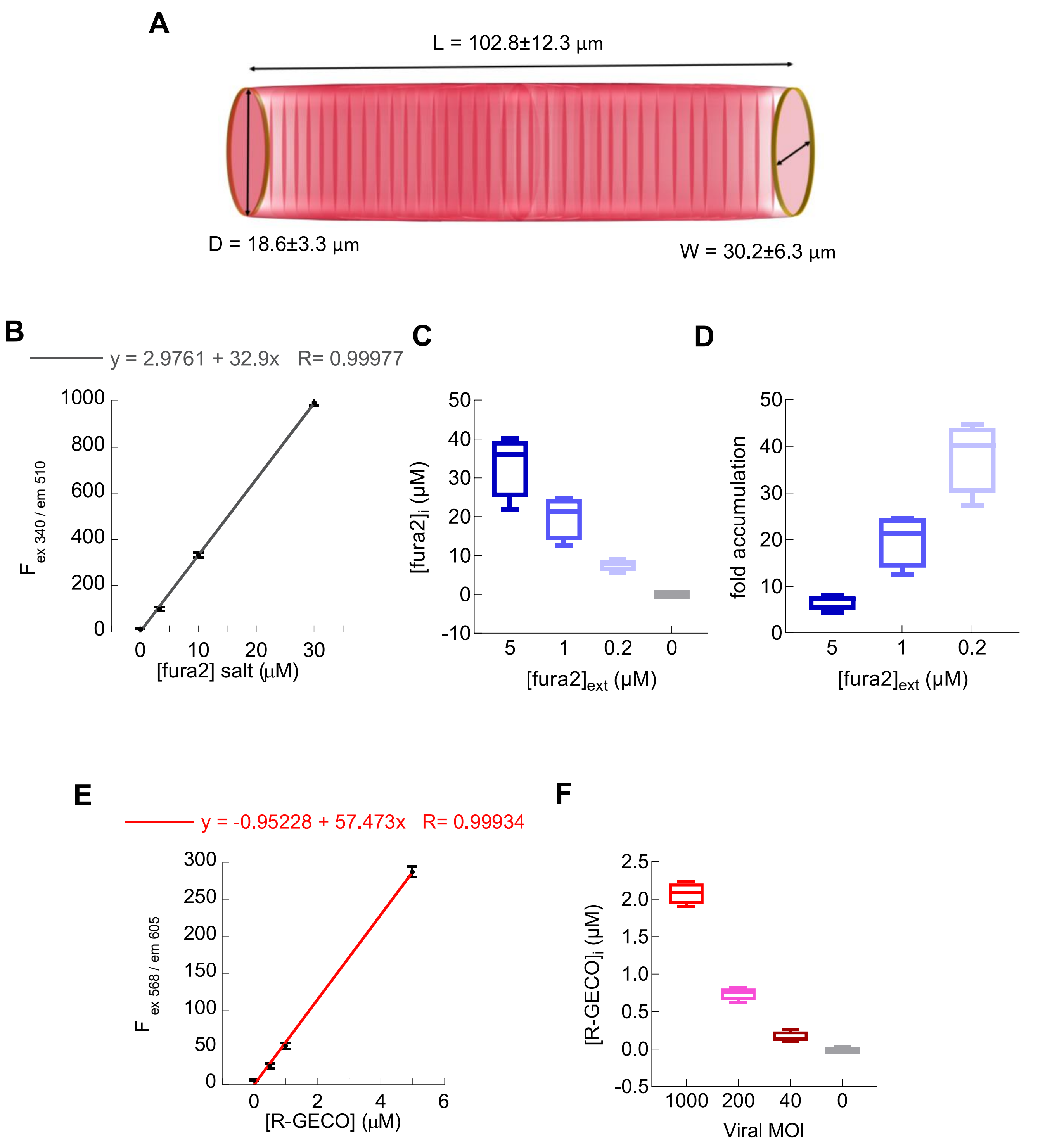

S5. Fura2-AM-ester accumulates in adult cardiomyocytes upon normal loading at intracellular concentrations significantly higher than adenovirally expressed R-GECO.

Confocal microscopy was used to determine the average cardiomyocyte size (A), and the brightness of fura2 fluorescence (emission at 510 nm, excitation of 340nm) was calculated using increasing concentrations of fura2 salt (B). These data were used to estimate the concentration of fura2 accumulated in cardiomyocytes under a range of external fura2-AM-ester loading concentrations (5, 1 and 0.2 μM respectively) following cell lysis in fura2 free PBS (C). The fold accumulation over the external loading concentration of fura2-AM-ester was subsequently calculated (D). For RGECO, the same cell dimensions were used in combination with the fluorescence emission at 605 nM with increasing concentrations of recombinant RGECO protein (E), to estimate the intracellular concentration of RGECO following incubation with increasing multiplicities of infection (MOI) of recombinant RGECO expressing adenovirus and cell lysis as with fura2 loaded cells (F). Box and whisker plots give the median average, interquartile range (box) and minimum and maximum data spread (whiskers) (*n*=4 cell lysate recordings for fura2 and R-GECO) .

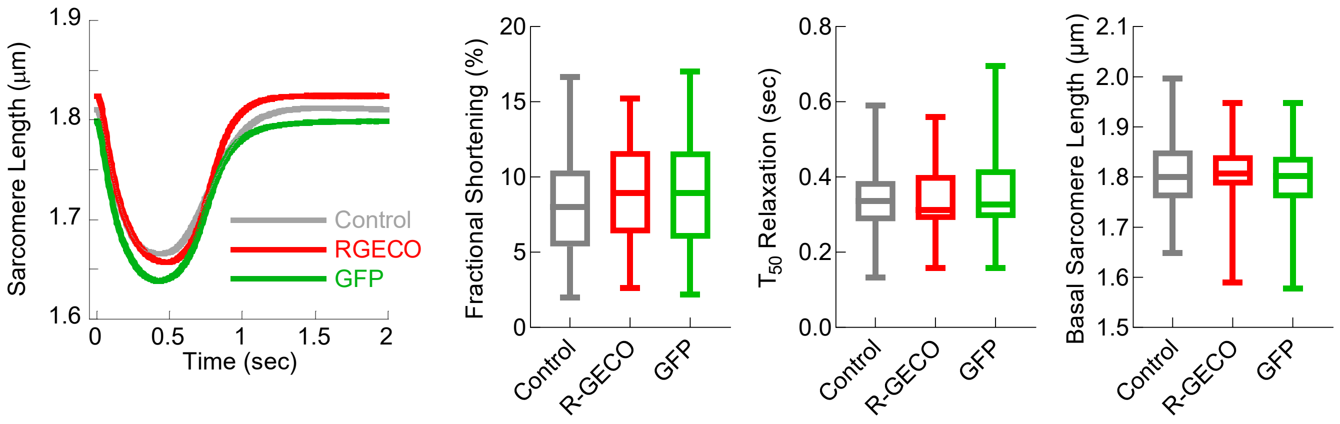

Fig. S6. Unloaded sarcomere shortening curves and extracted parameters to compare RGECO to uninfected and GFP control transduced guinea pig left ventricular cardiomyocytes.

Sarcomere shortening traces are averages from RGECO (*n*=52 cells from 3 separate isolations)(Red), uninfected control (*n*=52 cells from 3 separate isolations) (grey) and GFP transduced control (*n*=70 cells from 3 separate isolations) (green) guinea pig left ventricular cardiomyocytes. Extracted parameters show unchanged fractional shortening, relaxation time and basal sarcomere length (p>0.05). Box and whisker give the median average, interquartile range (box) and minimum and maximum data spread (whiskers).

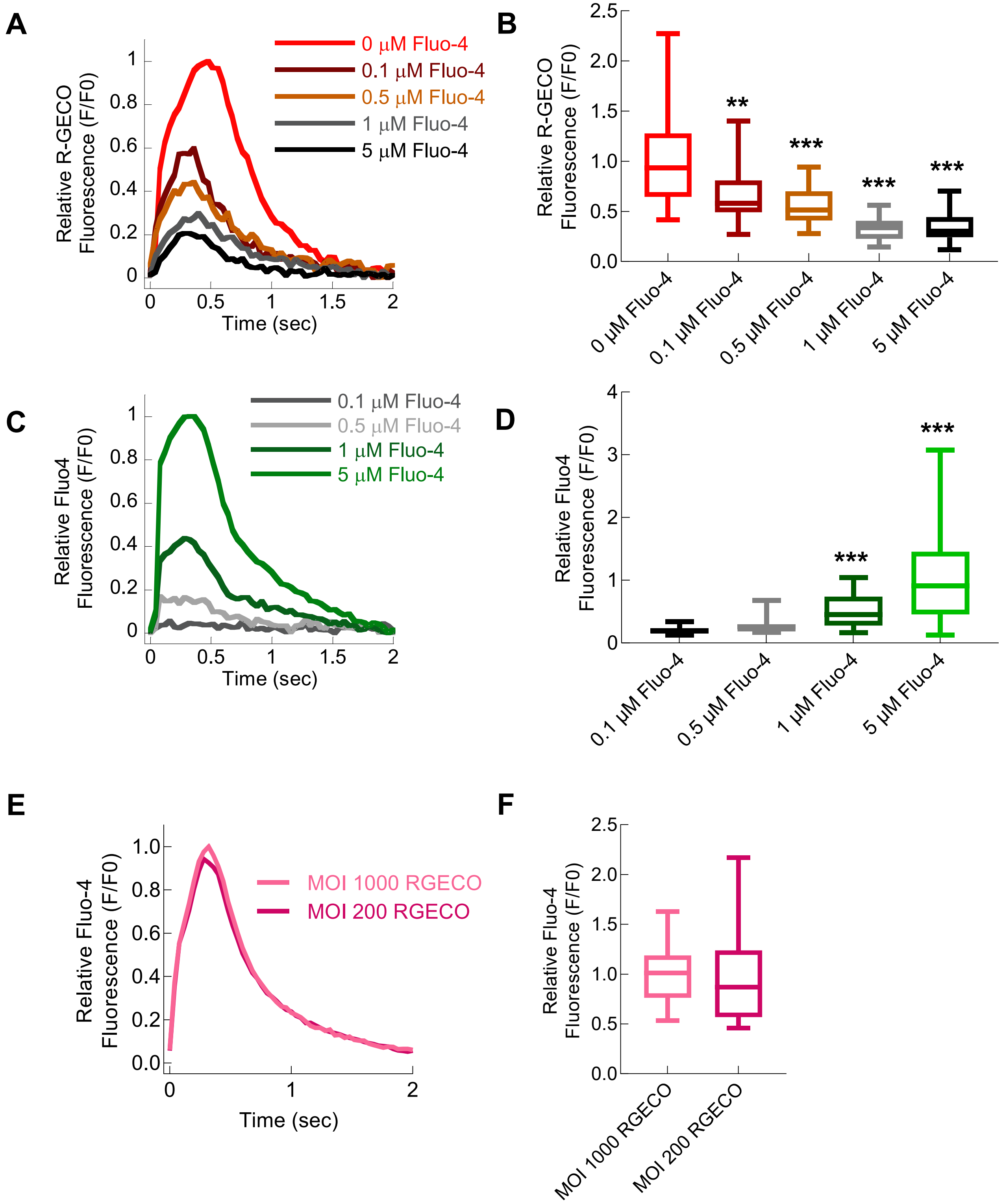

Fig. S7. The green chemical dye Fluo-4 reduces the fluorescence of R-GECO in a dose dependant manner during pairwise multicolour imaging.

Guinea pig left ventricular cardiomyocytes were transduced with recombinant adenovirus expressing R-RGECO at 400 MOI. Cells were split and incubated with 0.1, 0.5, 1 and 5 µM of Flou-4-AM ester for 5 minutes at room temperature. Loading with all concentrations of Fluo-4 significantly reduced RGECO Ca2+ transient amplitude indicating that Ca^{2+} is sequestered by the chemical dye Box and whisker plots B and D give the median average, interquartile range (box) and minimum and maximum data spread (whiskers). ** = p<0.01 and *** = p<0.001 using one way ANOVA, (*n*=23-48 cells from 3 separate isolations).

A

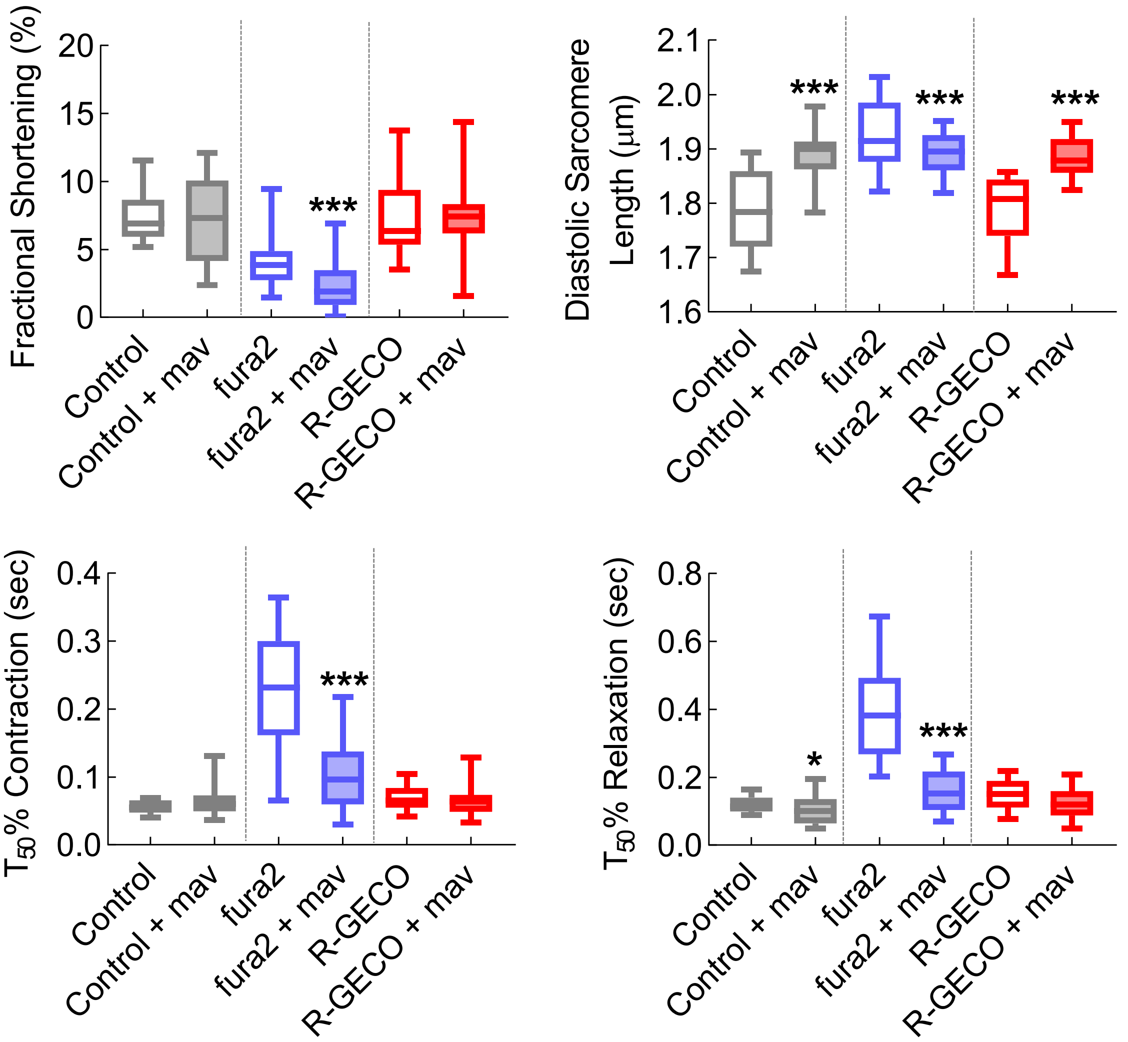

B

Sensor	Drug	*n*	Fractional Sarcomere Shortening (%)	Diastolic Sarcomere Length (µm)	Time to 50% Contraction (sec)	Time to 50% Relaxation (sec)
Control	**DMSO**	*26*	7.456±0.381	1.791±0.015	0.056±0.002	0.120±0.005
	**250 nM mavacamten**	*36*	7.177±0.500	**1.892±0.007 *****	0.063±0.004	**0.102±0.006 ***
1 µM fura2	**DMSO**	*20*	4.351±0.534	1.925±0.014	0.231±0.018	0.395±0.031
	**250 nM mavacamten**	*18*	**2.409±0.464 *****	**1.894±0.009 *****	**0.106±0.014 *****	**0.153±0.014 *****
R-GECO	**DMSO**	*22*	7.101±0.560	1.792±0.013	0.067±0.004	0.150±0.009
	**250 nM mavacamten**	*25*	7.206±0.532	**1.885±0.007 *****	0.066±0.005	0.125±0.009

Fig. S8. Extracted parameters from unloaded sarcomere shortening curves upon the application of 250 nM mavacamten.

Extracted values from paired experimental comparisons of mavacamten (mav) to control, were taken from unloaded sarcomere shortening in **Figure 6** and presented as box and whisker in A, and give the median average, interquartile range (box) and minimum and maximum data spread (whiskers), tabulated in B, (*n*=18-36 from 3 separate isolations). Significance values (highlighted red or blue (if directionally to opposite control experiments)) comparing pre and post treatment are p<0.001=*** and p<0.05=* using Mann-Whitney tests.

A

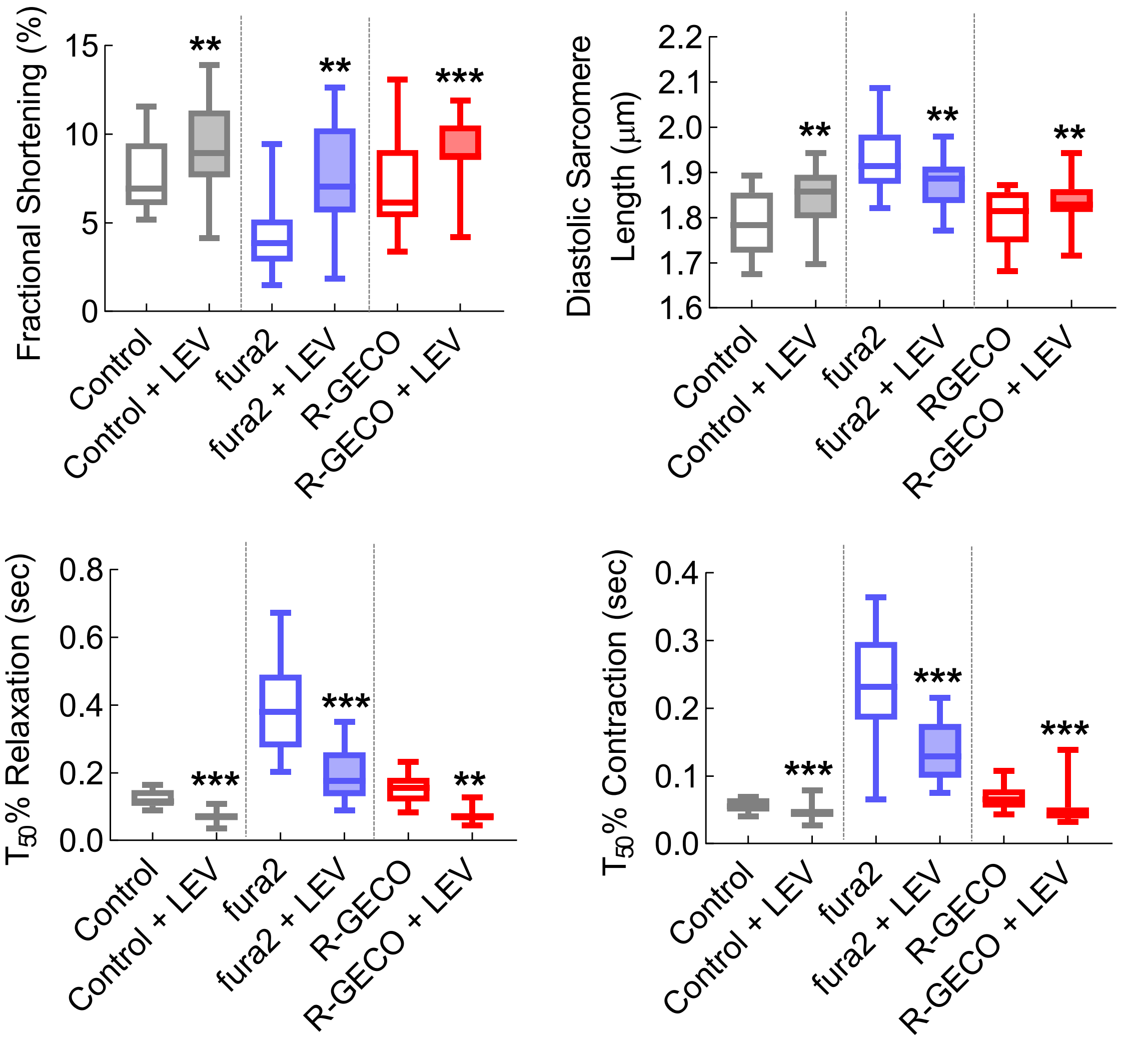

B

Sensor	Drug	*n*	Fractional Sarcomere Shortening (%)	Diastolic Sarcomere Length (µm)	Time to 50% Contraction (sec)	Time to 50% Relaxation (sec)
Control	**DMSO**	*24*	7.563±0.561	1.791±0.015	0.056±0.002	0.122±0.005
	**10 µM levosimendan**	*36*	**9.266±0.409 ****	**1.849±0.010 ****	**0.045±0.002 *****	**0.071±0.003 *****
**1 µM fura2**	**DMSO**	*22*	4.392±0.491	1.926±0.015	0.232±0.017	0.386±0.028
	**10 µM levosimendan**	*27*	**7.286±0.613 ****	**1.875±0.011 *****	**0.139±0.008 *****	**0.193±0.014 *****
R-GECO	**DMSO**	*29*	7.001±0.454	1.799±0.011	0.071±0.003	0.152±0.008
	**10 µM levosimendan**	*27*	**9.244±0.300 *****	**1.836±0.009 ****	**0.047±0.004 ****	**0.072±0.004 *****

Fig. S9. Extracted parameters from unloaded sarcomere shortening curves upon the application of 10 µM levosimendan. Extracted values from paired experimental comparisons of levosimendan (LEV) to control**,** were taken from unloaded sarcomere shortening in **Figure 6** and presented as box and whisker in A, and give the median average, interquartile range (box) and minimum and maximum data spread (whiskers), tabulated in B, (*n*=22-36 from 3 separate isolations). Significance values (highlighted red or blue (if directionally to opposite control experiments)) comparing pre and post treatment are p<0.001=*** and p<0.01=** using Mann-Whitney tests.

A

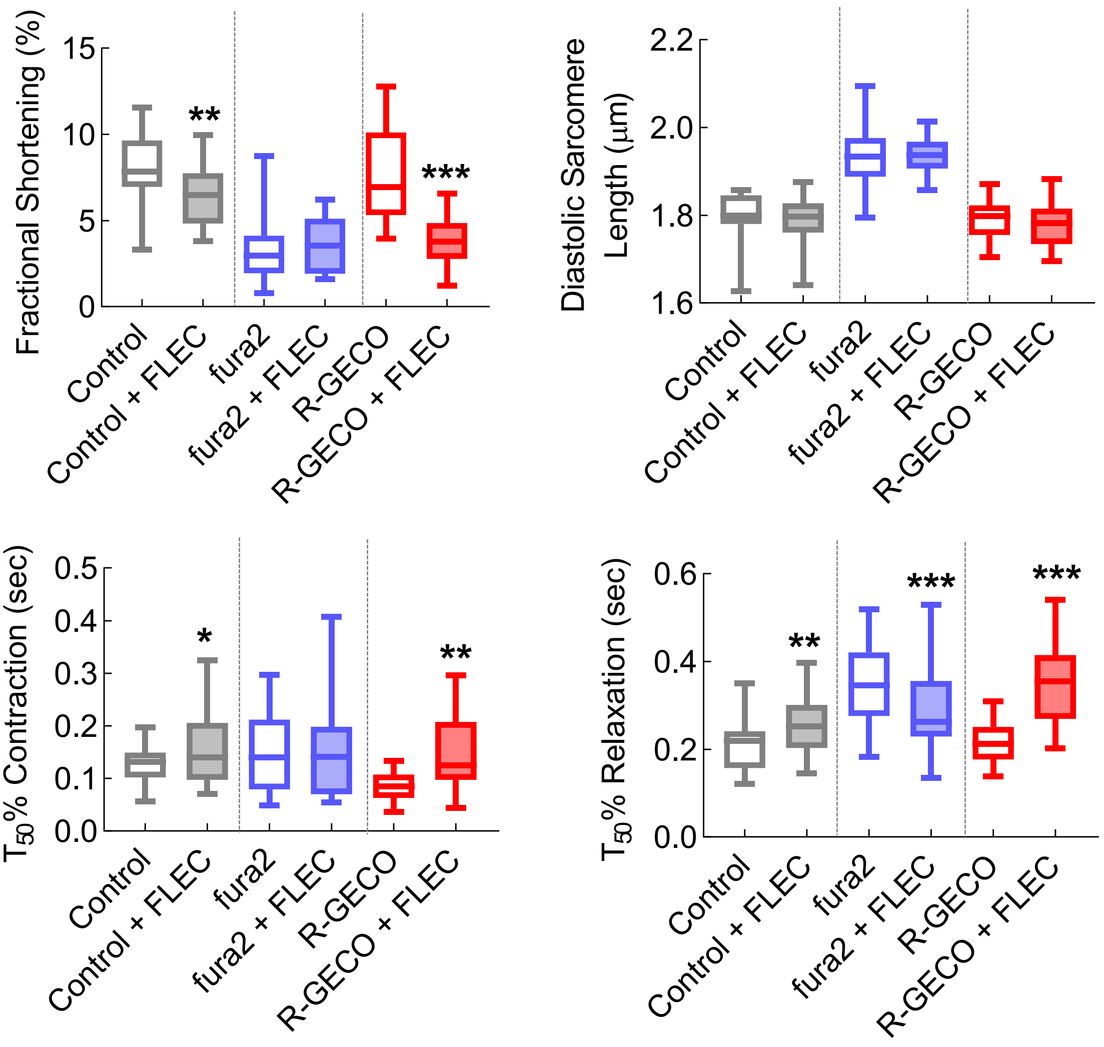

B

Sensor	Drug	*n*	Fractional Sarcomere Shortening (%)	Diastolic Sarcomere Length (µm)	Time to 50% Contraction (sec)	Time to 50% Relaxation (sec)
Control	**DMSO**	*26*	8.094±0.398	1.799±0.010	0.125±0.007	0.211±0.011
	**0.5 mM flecanide**	*24*	**6.404±0.354 ****	1.789±0.012	**0.160±0.014 ***	**0.262±0.015 ****
**1 µM fura2**	**DMSO**	25	3.274±0.388	1.932±0.013	0.150±0.015	0.348±0.019
	**0.5 mM flecanide**	22	3.539±0.343	1.926±0.015	0.157±0.021	**0.293±0.022 *****
**R-GECO**	**DMSO**	25	7.715±0.556	1.791±0.009	0.084±0.006	0.217±0.010
	**0.5 mM flecanide**	26	**3.798±0.272 *****	1.778±0.010	**0.166±0.027 ****	**0.348±0.016 *****

Fig. S10. Extracted parameters from unloaded sarcomere shortening curves upon the application of 0.5 µM flecainide. Extracted values from paired experimental comparisons of flecainide (FLEC) to control**,** were taken from unloaded sarcomere shortening in **Figure 6** and presented as box and whisker in A, and give the median average, interquartile range (box) and minimum and maximum data spread (whiskers), tabulated in B, (*n*=22-26 from 3 separate isolations). Significance values (highlighted red or blue (if directionally to opposite control experiments)) comparing pre and post treatment are p<0.001=***, p<0.01=** and p<0.05=* using Mann-Whitney tests.

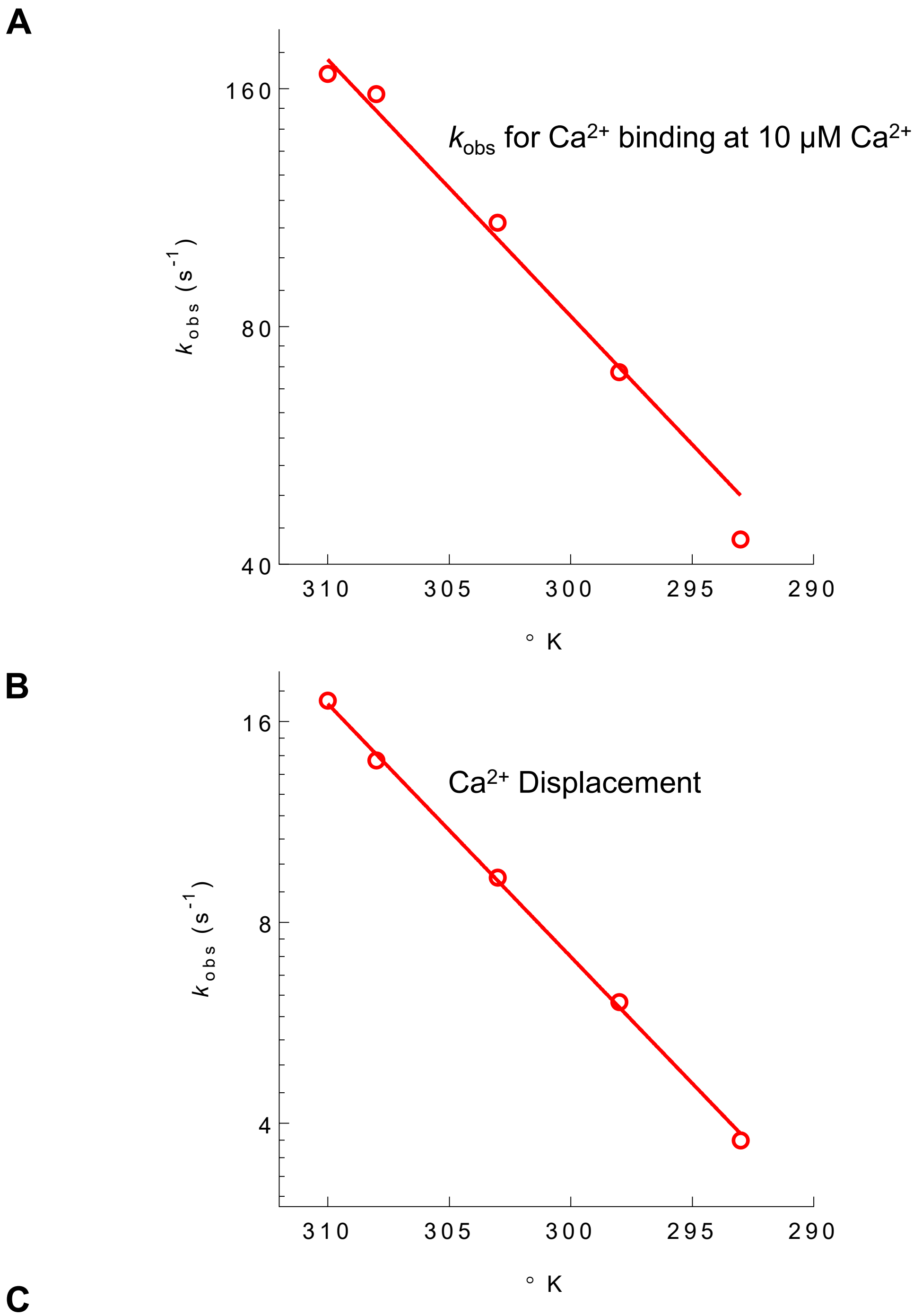

C

	**k_{off}/s^{-1}**		**$k_{on}/M^{-1}s^{-1}$**	
**Temp (°C)**	**25**	**37**	**25**	**37**
R-GECO	6.1	17.2	2.1×10^{-4}	4.59×10^{-4}
fura2	96.8		6.02×10^{-8}	

Fig. S11. Kinetic determination of R-GECO k_{on} and k_{off} by stopped flow.
Arrhenius plots of the observed rate constant of Ca^{2+} binding (A) and Ca^{2+} release rate constant (B) as determined by stopped flow measurement of 0.125 μM purified protein. Data for room (25°C) and body (37°C) temperature are shown in C.

Sensor	Drug	*n*	fura2 Fluorescence Amplitude ($F_{365/380}$) / GECO Peak Intensity ($\Delta F_{581}/F$)	Time to 50% Ca^{2+} Binding (sec)	Time to 50% Ca^{2+} Release (sec)	Basal Fluorescence ($F_{365/380}$)
1 μM fura2	**DMSO**	*22*	0.282±0.019	0.035±0.005	0.278±0.014	1.091±0.026
	**250 nM mavacamten**	*27*	0.270±0.016	0.033±0.003	**0.213±0.020 ***	**1.297±0.020 *****
R-GECO	**DMSO**	*79*	11.89±0.697	0.077±0.003	0.342±0.007	-
	**250 nM mavacamten**	*84*	**10.85±0.438 ***	0.074±0.003	**0.227±0.006 *****	-
1 μM fura2	**DMSO**	*22*	0.286±0.018	0.035±0.005	0.287±0.015	1.105±0.026
	**10 μM levosimendan**	*32*	**0.397±0.019 *****	0.038±0.003	**0.230±0.008 ****	1.146±0.022
R-GECO	**DMSO**	*77*	10.54±0.534	0.078±0.003	0.329±0.008	-
	**10 μM levosimendan**	*83*	**14.17±0.519 *****	0.071±0.003	**0.245±0.004 *****	-
1 μM fura2	**DMSO**	24	0.257±0.027	0.037±0.004	0.302±0.023	1.086±0.018
	**0.5 μM Flecanide**	24	**0.161±0.013 *****	0.039±0.006	**0.377±0.027 ***	**1.161±0.012 *****
R-GECO	**DMSO**	50	10.25±0.790	0.074±0.004	0.276±0.007	
	**0.5 μM Flecanide**	52	11.54±0.552	0.074±0.003	**0.316±0.007 *****	

Table. S1. Extracted parameters from Ca^{2+} transient measurements.
Extracted values from paired experimental comparisons of mavacamten, levosimendan and flecainide to control, were taken from Ca^{2+} transients in Fig.7 , (*n*=22-84 from 3 separate isolations). Significance values (highlighted in red) comparing pre and post treatment are p<0.001=***, p<0.01=** and p<0.05=* using Mann-Whitney tests.

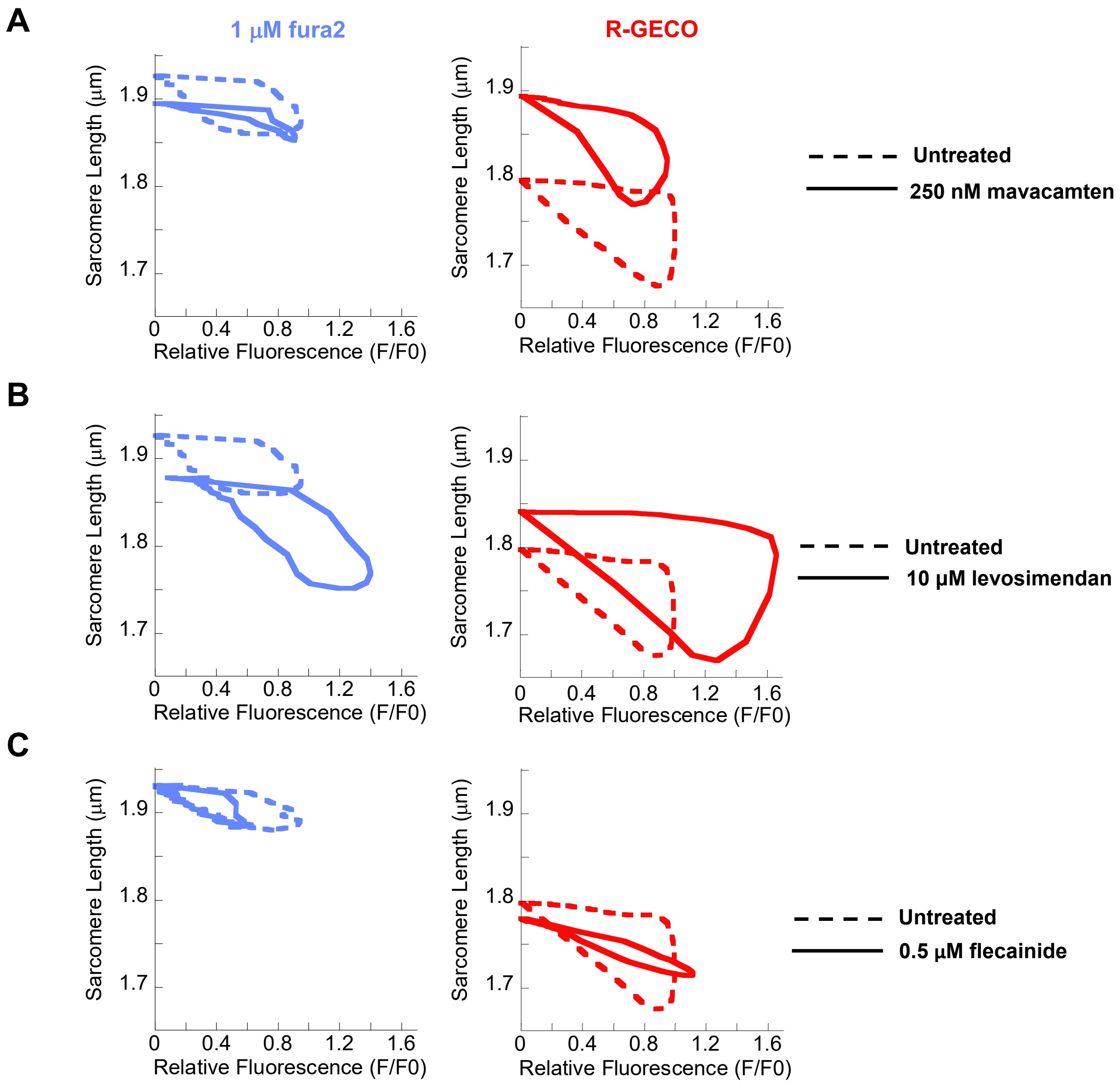

Fig. S12. Contractility/Ca^{2+} loops of guinea pig cardiomyocytes loaded with fura2 or R-GECO in the presence of vehicle or drug (mavacamten, levosimendan or flecainide).

Averaged loops from control (dashed line) or drug exposed (solid line) are given for each of the stated indicator, and small molecule combinations mavacamten (A), levosimendan (B) and flecainide (C). Due to variability inherent in primary cell preparations controls are presented for each condition and the sample split for paired analysis with drug.

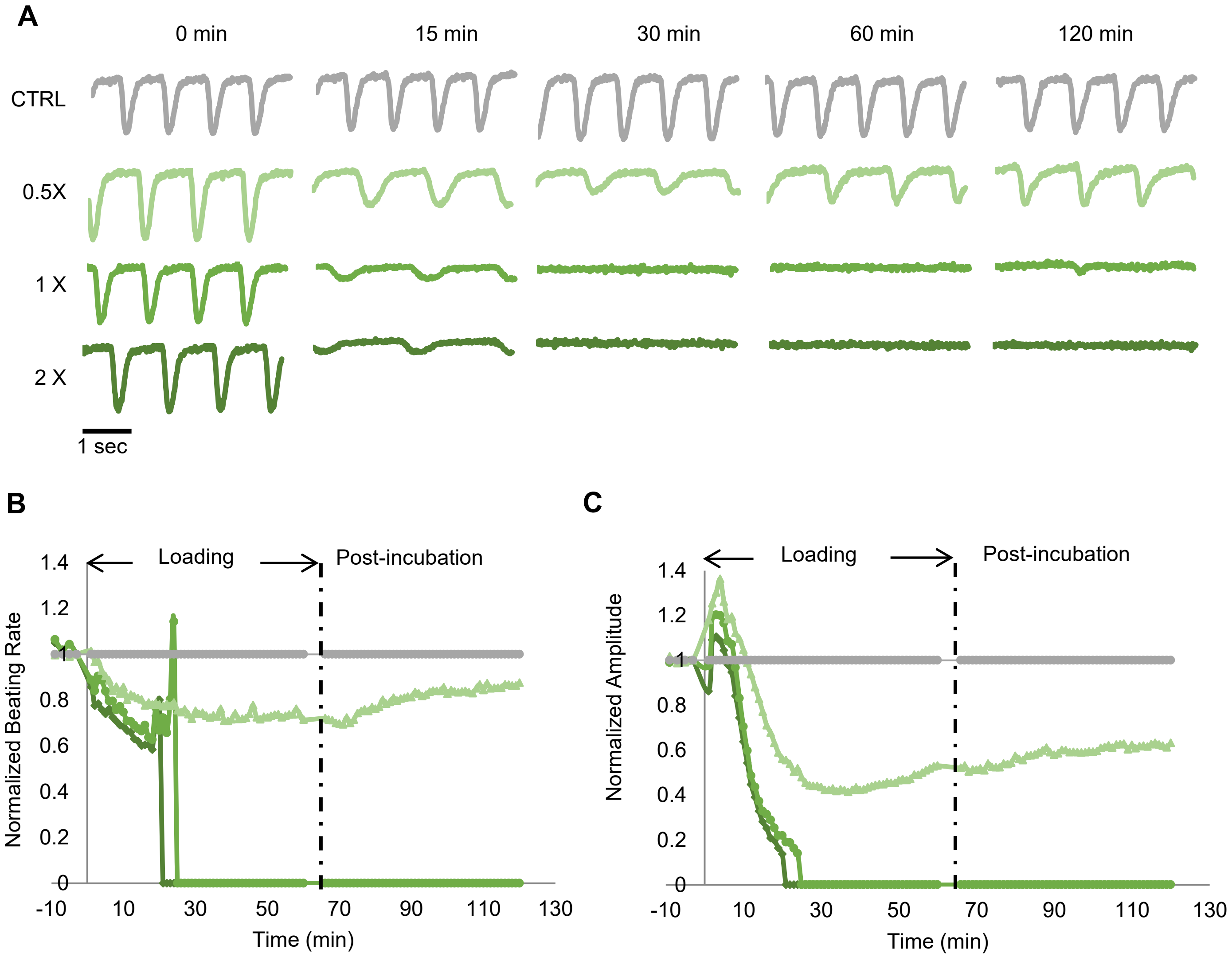

Fig. S13. The Ca^{2+} dye FLIPR Calcium 5 reduces the contractile activity of hiPSC derived cardiomyocytes. Label-free impedance monitoring to quantitatively evaluate hiPSC cardiomyocyte movement, was undertaken with the Xcelligence RTCA Cardio system using 20,000 CDI cells in the presence of serial dilutions of the green Ca^{2+} dye FLIPR 5. A shows the representative data windows of the impedance trace are shown for the one-hour dye loading and subsequent one hour observation period. B and C are the data extracted for beat rate, and beat amplitude show that dye incubation reduces the measured beat rate and beat amplitude before evidence of contractility is irreversibly lost. Dye doses around the recommended final 1x concentration are shown as increasingly dark green lines in A, B and C, the vehicle treated control is in grey.
